# Genome-wide comparative analysis of homeobox transcription factors in rice and expression profiles of selected genes reveal potential tissue and developmental stage specificity as well as abiotic stress responsiveness

**DOI:** 10.3389/fpls.2026.1859527

**Published:** 2026-10-27

**Authors:** Libia Iris Trejo-Téllez, José Ignacio Saavedra-Ávila, Hugo Fernando Escobar-Sepúlveda, Fernando Carlos Gómez-Merino

**Affiliations:** 1 Department of Soil Sciences, College of Postgraduates in Agricultural Sciences, Texcoco, Mexico; 2 Department of Plant Physiology, College of Postgraduates in Agricultural Sciences, Texcoco, Mexico; 3 Department of Genetics, College of Postgraduates in Agricultural Sciences, Texcoco, Mexico; 4 Facultad de Ingeniería, Universidad Católica del Maule, Talca, Chile

**Keywords:** drought, eFP browser, gene expression profiling, *Oryza sativa*, osmotic stress, Poaceae, salinity

## Abstract

**Introduction:**

Homeobox (HB) transcription factors are essential for plant development and can participate in regulating plant responses to diverse biotic and abiotic stresses.

**Methods:**

In this study, we performed an *in-silico* analysis of the different HB gene and protein subfamilies in rice, including *Oryza sativa* subsp. *indica* and *O. sativa* subsp. *japonica*.

**Results:**

A total of 112 and 156 sequences annotated as HB transcription factors were retrieved from PlantTFDB for *O. sativa* subsp. *indica* and subsp. *japonica*, respectively, which showed 94% sequence identity. The homeobox–plant homeodomain finger (HB-PHD) subfamily showed 100% identity, whereas the three-amino-acid-loop-extension (TALE), WUSCHEL-related homeobox (WOX), and zinc-finger-homeodomain (ZF-HD) subfamilies displayed values equal to or higher than 95%; in contrast, HB-OTHERS showed the lowest identity (82%). Phylogenetic analysis grouped the proteins into six major subfamilies: HB-PHD, HD-ZIP, TALE, WOX, ZF-HD, and HB-OTHERS. In the promoter regions of *HB* genes of *O. sativa* subsp. *japonica*, 688 *cis*-regulatory motifs were retrieved as potentially associated with hormonal and stress responses such as drought, salinity, and cold; among the most frequent were ABRE, CGTCA-motif, TGACG-motif, and ARE. In addition, *de novo* motif analysis retrieved regulatory patterns specific to *HB* genes with significant similarity to transcription factors reported in specialized databases, suggesting conserved regulatory mechanisms. Selected representative genes from the *TALE*, *WOX*, and *HD-ZIP* subfamilies displayed relatively high expression under salinity or drought conditions.

**Discussion:**

Overall, our findings expand the understanding of the diversity and potential functions of HB transcription factors in rice, providing a comparative framework and a set of candidate *HB* genes for subsequent experimental studies addressing their roles in rice development and abiotic-stress responses.

## Graphical Abstract

**Figure:**
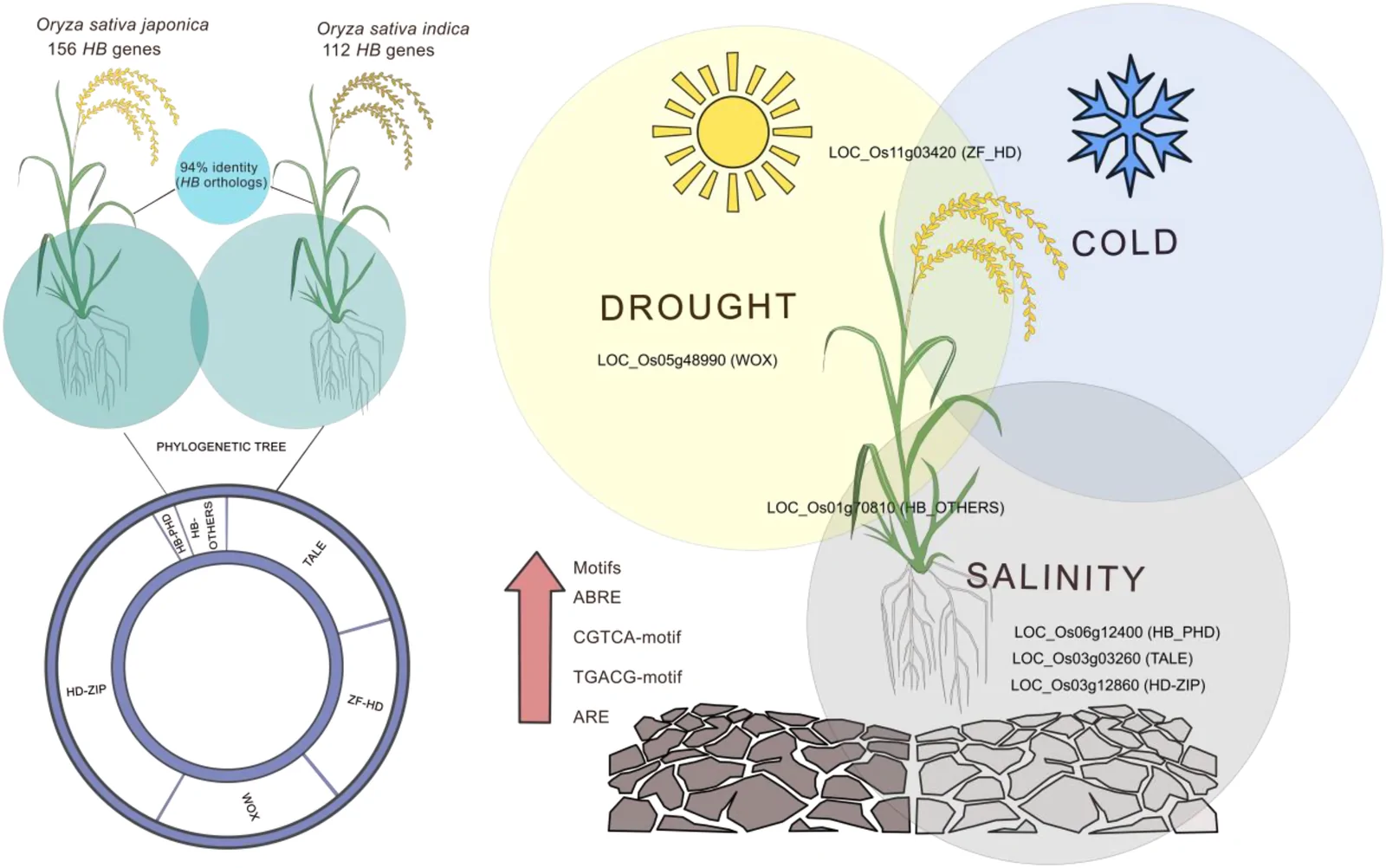

## 1 Introduction

*Homeobox* (*HB*) genes constitute a large and diverse superfamily of genes encoding transcription factors that harbour a typical homeodomain (HD), which is a conserved ∼ 60-amino acid motif found in most if not all eukaryotic organisms (Mukherjee et al., 2009; Holland, 2013; Khan et al., 2018; Hou et al., 2021; Lewin et al., 2021; Pongpom et al., 2024; Gao et al., 2025). According to Bürglin and Affolter (2016), there are 16 major classes of HB transcription factors in animal cells: ANTP, PRD, PRD-LIKE, POU, HNF, CUT (with four subclasses: ONECUT, CUX, SATB, and CMP), LIM, ZF, CERS, PROS, SIX/SO, and the TALE superclass with the classes IRO, MKX, TGIF, PBC, and MEIS. In plant cells, *HB* genes have been differentiated into 14 distinct classes with various structures characterized by conserved intron–exon structure and a unique domain architecture, including HD-ZIP I to IV, WOX, NDX, PHD, PLINC, LD, DDT, SAWADEE, PINTOX, and the two TALE classes KNOX and BEL (Mukherjee et al., 2009; Xu et al., 2019). Most of these classes encode additional domains apart from the homeodomain (Holland, 2013), thus providing further DNA specificity for the regulation of a plethora of biological processes related to plant morphogenesis, growth, development, and responses to environmental cues (Haecker et al., 2004; Zhang et al., 2012; Chew et al., 2013; Peterson et al., 2013; Bhattacharjee et al., 2016; Perotti et al., 2017; Li et al., 2022). The highly conserved HD motif in HB transcription factors folds into a characteristic DNA-binding structure composed of three alpha helices, of which the second and third form a helix-turn-helix motif (Bürglin and Affolter, 2016). The HD motif can bind to *cis*-acting elements of their target genes. Most HD transcription factors are able to bind DNA as monomers with high affinity, through interactions made by helix III (the so-called recognition helix) and a disordered N-terminal arm located beyond helix I. The highly conserved HD motif among diverse transcription factors suggests that this structure is crucial in preserving HB functionality and that this domain plays key roles in the regulation of vital processes of plants (Ariel et al., 2007; Mukherjee and Brocchieri, 2010), including the responses to osmotic stress triggered by cold, salinity, or drought (Hou et al., 2021; Li et al., 2022; Xu et al., 2023). Interestingly, *HB* genes have been found to also be associated with hormonal pathways in plants. For instance, *HD-ZIP* genes may modulate responses to hormones such as auxins, cytokinins, abscisic acid, and brassinosteroids (Sessa et al., 2018, 2024). Other *Homeobox* gene types, such as *KNOX*, are involved in the regulation of hormone-dependent processes by modulating hormonal balance in the meristem (Hay et al., 2004). Consistently, the *WOX* gene subfamily in rice (*Oryza sativa* L.) has been shown to play an important role in the development of multiple plant organs; moreover, several of its members respond directly to hormonal signals such as auxins, cytokinins, and gibberellins, and are activated under abiotic stress conditions including drought, salinity, and cold (Cheng et al., 2014; Wang et al., 2024; Deng et al., 2025). Therefore, *Homeobox* genes may integrate environmental and hormonal signals to regulate plant development and adaptation to diverse stress conditions.

Drought has affected more people worldwide in the last 50 years than any other natural hazard. According to the United Nations, more than two billion hectares of previously productive land are degraded, while 12 million hectares of land capable of producing 20 million tons of grain are lost due to drought and desertification every year (United Nations, 2022; UNEP-WCMC, 2020). Climate change is expected to exacerbate the risk of droughts in many vulnerable regions of the world, particularly those with rapid population growth, vulnerable populations, and challenges regarding food security (CRED and UNDRR, 2020; Li et al., 2026). In addition, salt and cold stress, which cause osmotic stress, are also being aggravated by the impact of climate change on agriculture (Liaqat et al., 2025; Wu et al., 2026).

To meet these challenges, investigating the molecular mechanisms of plant responses to the osmotic stress imposed by drought is a pressing issue. Furthermore, developing an integrated research framework combining physiology, biochemistry, genomics, systems biology, modelling, and breeding to improve plant responses to drought under a climate change scenario remains a daunting task. Since climate change is widespread and intensifying, more rapid, abrupt, and severe effects of drought are to be expected. Consequently, agriculture and food production must be prepared for a more uncertain future; therefore, a better understanding of the effect of frequent and more extreme drought stress on physiological, biochemical, and molecular processes in plants is needed.

It has been largely demonstrated that HB transcription factors are responsive to osmotic stress, including water deficit, salinity, and low temperatures. In pepper (*Capsicum annuum* L.), the *dehydration-responsive homeobox 1* gene, *CaDRHB1*, plays a positive role in the dehydration response (Lim et al., 2018). In grapevine (*Vitis vinifera* L.), the expression of the *HB12* transcription factor was upregulated in response to water stress (Catacchio et al., 2019). In the hybrid poplar 84K (*Populus alba*×*Populus glandulosa*), the *WUSCHEL-related homeobox* gene *PagWOX11/12a* was induced in response to drought stress, enhancing root elongation and biomass growth (Wang et al., 2020a), through the modulation of reactive oxygen species scavenging (Liu et al., 2021b). In sesame (*Sesamum indicum* L.), the genes *SiHZ7* and *SiHZ35* were significantly induced by drought stress, and may work in a coordinated fashion by changing their gene expression levels to fine-tune processes associated with growth (early seedling stage) and water stress (Mehmood et al., 2022). In soybean [*Glycine max* (L.) Merr.], at least three *HB-PHD* genes were upregulated upon drought stress (Xuan et al., 2022). In alfalfa (*Medicago sativa* L.), various *WUSCHEL-related homeobox* (*WOX*) genes showed responsiveness to dehydration artificially induced by applying polyethylene glycol 6000 (PEG 6000) (Xu et al., 2023). In rapeseed (*Brassica napus* L.), Żyła et al. (2024) analyzed the functional and regulatory diversity of homeobox-leucine zipper transcription factors *BnaHB6* under dehydration and salt stress, reporting that four *BnaHB6* homologues have distinct functions in response to stress despite high sequence similarity, possibly indicating different binding preferences with *BnaABF4* and *BnaDREB2A*. Khan et al. (2024) described the multifaceted functions of *WOXs* in complex developmental and abiotic stress-sensing networks, with particular emphasis on regulating the related genes and other transcription factors, suggesting that *WOXs* are essential components of the gene regulatory networks involved in plant responses to drought, salinity, freezing, and heat-induced stress.

Belonging to the Poaceae family, cereal crops such as maize (*Zea mays* L.), wheat (*Triticum aestivum* L.), and rice are vital to the food supply for the increasing human population because they are dense sources of calories and nutrients, have a long shelf life, and are adaptable to diverse climates. Globally, just these three crops provide more than 50% of the world’s total food energy (OECD-FAO, 2025). Rice, a staple food for over half the global population, ranks third in cereal production just after maize and wheat, with over 557 million tons in 2025 (FAO, 2025). However, rice is highly vulnerable to climate change due to drought, flooding, and extreme temperatures, which disrupt its physiology and reduce yields and crop quality (Algarni et al., 2025). Therefore, a better understanding of stress tolerance is crucial for identifying specific physiological, biochemical, and molecular mechanisms to improve resilience in rice, and promoting sustainable agricultural practices in environments exposed to limited water resources, drought episodes, and other abiotic stress factors.

Within the Poaceae family, the sorghum [*Sorghum bicolor* (L.) Moench] *HB* genes *PTSb00175.1* and *PTSb00173.1* displayed down-regulated expression in plants exposed to mild and severe moisture stress (Aglawe et al., 2012). In rice, the *WUSCHEL-related homeobox* gene *WOX11* increased drought resistance by controlling root hair formation and root system development (Cheng et al., 2016), while *OsWOX13* enhanced drought tolerance and triggered early flowering (Minh-Thu et al., 2018). In a meta-analysis of seven rice drought-tolerant genotypes, 20 *HB* genes displayed upregulation upon drought stress, while 18 were downregulated (Sircar and Parekh, 2019). In wheat, the *TALE* gene *TaKNOX11-A* confers tolerance against drought and salt stress by decreasing malondialdehyde (MDA) and increasing proline contents (Han et al., 2022). In sorghum, the *TALE* genes *SbTALE02* and *SbTALE12* exhibited enhanced expression in plants exposed to osmotic stress induced by polyethylene glycol (PEG), while *SbTALE23* was transcriptionally active under methyl jasmonate (MeJA) and salicylic acid (SA) treatments (Yao et al., 2024).

In a previous genome-wide analyses of *Homeobox* (*HB*) genes, Jain et al. (2008) identified a total of 107 *HB* genes in the rice (*Oryza sativa* subsp. *japonica* cv. Nipponbare) genome, and grouped them into 10 distinct subfamilies based upon their domain composition and phylogenetic analysis. A large number of *HB* genes were found to be duplicated, which suggests segmental duplications in rice. *HB* gene expression during various stages of panicle and seed development were identified, while others were differentially expressed under various abiotic stress conditions. In barley (*Hordeum vulgare*), Zhang et al. (2025b) performed a genome-wide identification of the *WUSCHEL-Related Homeobox* (*WOX*) gene subfamily, and revealed a potential role of *HvWOX8* in salt tolerance. They identified 12 *HvWOX* genes assigned from chromosome 1 to chromosome 5, which were highly expressed in reproductive tissues such as anthers or ovaries. *Cis*-element analyses suggested that there were multiple stress- and hormone-responsive elements in the *HvWOX* gene promoters. In sugarcane (*Saccharum* spp. hybrids), Chen et al. (2025) carried out a genome-wide identification of 66 *Homebox* (*ScHB*) genes, with *ScHB56* strongly associated with tillering via GWAS analysis. Just recently, Alshamrani and Safhi (2026) conducted a genome-wide identification and characterization of the *HB-Others* gene subfamily in *Panicum hallii*, examining gene structure, conserved domains, physicochemical properties, phylogenetic relationships, and promoter *cis*-regulatory elements, while revealing distinct tissue-dependent transcriptional responses under salinity stress. Although various genome-wide analyses exploring *HB* genes in rice and other grasses have been conducted, comprehensive bioinformatic assessments exploring their potential expression dynamics, hormonal regulation, and functional roles in abiotic stress responses based on microarray and RNA-seq data are lacking. Thus, herein we performed an integrative bioinformatic analysis including: comparison of the number of *HB* genes among *Oryza* species, with an emphasis on *O. sativa* subsp. *indica* and subsp. *japonica*; identification of best-hit homologs between the two subspecies using BLASTp; joint phylogenetic analysis of HB proteins; analysis of promoters, predicted *cis*-regulatory elements, and *de novo* discovered motifs; and examination of public expression profiles for selected representative genes under abiotic stress and across different tissues or developmental stages.

## 2 Materials and methods

### 2.1 Data sources and retrieval of predicted HB protein sequences

Protein sequences annotated as Homeobox transcription factors in *Oryza sativa* subsp. *indica* and *O. sativa* subsp. *japonica* were retrieved from PlantTFDB v5.0 (http://planttfdb.gao-lab.org/index.php) (Jin et al., 2014, 2017). Initially, a total of 268 HB protein sequences were downloaded from PlantTFDB: 112 from *Oryza sativa* subsp. *indica* and 156 from subsp. *japonica*. To avoid redundancy caused by multiple isoforms, only version 1 (.1) of each gene was retained. Following this filtering process, a final set of 223 sequences (112 from *indica* and 111 from *japonica*) was obtained for phylogenetic analysis. Family assignments and gene identifiers provided by PlantTFDB were retained for the initial dataset.

For promoter extraction and chromosomal mapping of *O. sativa* subsp. *japonica* cv. Nipponbare, the genome sequence and corresponding GFF3 annotation from the MSU/Phytozome v7.0 assembly were downloaded from Phytozome (https://phytozome-next.jgi.doe.gov/info/Osativa_v7_0/) (Ouyang et al., 2007). Gene identifiers were cross-referenced between PlantTFDB and the genome annotation before downstream analyses.

### 2.2 Identification of best-hit homologs between *O. sativa* subspecies

A homology analysis was performed among the different HB protein subfamilies of *Oryza sativa* subsp. *indica* (query) and *O. sativa* subsp. *japonica* (reference/subject) using BLASTp (version 2.12) in an Ubuntu computational environment (Johnson et al., 2008). First, a BLAST database was constructed using the protein sequences of each HB transcription factor family from *O. sativa* subsp. *japonica*, as it provides a high-quality, well-annotated genome suitable for downstream analyses. Subsequently, FASTA protein sequence files from *O. sativa* subsp. *indica* were used as queries to identify their homologs in *O. sativa* subsp. *japonica*. The analysis was conducted using an *E*-value threshold of 1e^−5^ (0.00001) to ensure high alignment reliability, retaining only the best hit for each query sequence.

To provide independent validation of the HB protein classification based on conserved domain analysis, the 223 protein sequences included in the phylogenetic analysis were analyzed using InterProScan v5.77-108.0 (Jones et al., 2014). Searches were performed against Pfam v38.1, SMART v9.0, Conserved Domain Database (CDD) v3.21, and SUPERFAMILY v1.75. Identified matches were linked, where available, to their corresponding integrated InterPro annotations and Gene Ontology terms. All analyses were performed locally using the same configuration. For each protein, the source database, domain or family identifier and description, coordinates within the sequence, *E*-value or score, and corresponding InterPro annotation were recorded. These results were used to evaluate the concordance between the conserved domain architecture and the phylogenetic classification of the HB proteins.

### 2.3 Multiple sequence alignment and phylogenetic analysis

For this analysis, we used the protein sequences of HB transcription factors from rice subsp. *indica* and subsp. *japonica* (**Table 1**). The sequences were retrieved from the Plant Transcription Factor Database version 5.0 (PlantTFDB; https://planttfdb.gao-lab.org/) (Jin et al., 2017; Tian et al., 2020). A combined dataset of 223 *Oryza* HB transcription factor protein sequences, grouped into six families (HB-PHD, HD-ZIP, TALE, WOX, ZF-HD, and HB-OTHERS), was prepared in FASTA format and aligned using MUSCLE in MEGA 12 with default parameters (Kumar et al., 2024). The phylogenetic tree was inferred by the maximum likelihood (ML) method using IQ-TREE v2.4.0 (https://iqtree.github.io/), applying the JTT substitution model and 1, 000 bootstrap replicates (Nguyen et al., 2015; Minh et al., 2020). The resulting tree was uploaded to the iTOL platform (https://itol.embl.de/) for visualization and annotation (Letunic and Bork, 2024), using a six-color palette to distinguish each gene subfamily.

**Table 1:** Best-hit homology among Homeobox (HB) proteins per subfamily predicted in the proteome of *Oryza sativa* subsp. *indica* and *Oryza sativa* subsp. *japonica*.

Number	HB Subfamily	*Oryza sativa* subsp. *indica*	*Oryza sativa* subsp. *japonica*	Coverage (%)	*E*-value	Identity (%)
1	HB-PHD	BGIOSGA022583-PA	LOC_Os06g12400.1	93	0	100
1	HB-OTHERS	BGIOSGA004179-PA	LOC_Os01g48170.1	100	1.70E-175	100
2	HB-OTHERS	BGIOSGA005119-PA	LOC_Os01g70810.1	100	0	93
3	HB-OTHERS	BGIOSGA007535-PA	LOC_Os02g05450.1	100	0	100
4	HB-OTHERS	BGIOSGA013634-PA	LOC_Os03g55990.1	54	1.16E-142	86
5	HB-OTHERS	BGIOSGA014307-PA	LOC_Os07g24350.1	47	9.83E-18	41
6	HB-OTHERS	BGIOSGA019308-PA	LOC_Os05g09630.1	66	0	92
7	HB-OTHERS	BGIOSGA019617-PA	LOC_Os05g25600.1	62	1.59E-68	85
8	HB-OTHERS	BGIOSGA021223-PA	LOC_Os06g29020.1	55	4.52E-174	97
9	HB-OTHERS	BGIOSGA022450-PA	LOC_Os05g48820.1	98	0	99
10	HB-OTHERS	BGIOSGA023211-PA	LOC_Os06g39906.1	100	0	100
11	HB-OTHERS	BGIOSGA024401-PA	LOC_Os07g24350.1	58	2.31E-67	89
12	HB-OTHERS	BGIOSGA026156-PA	LOC_Os05g48820.1	31	1.05E-37	30
13	HB-OTHERS	BGIOSGA031128-PA	LOC_Os07g24350.1	21	5.88E-09	47
14	HB-OTHERS	BGIOSGA032532-PA	LOC_Os03g55990.1	9	4.67E-20	92
1	HD-ZIP	BGIOSGA000239-PA	LOC_Os02g35770.1	6	4.91E-21	55
2	HD-ZIP	BGIOSGA000782-PA	LOC_Os01g55549.1	100	0	92
3	HD-ZIP	BGIOSGA001131-PA	LOC_Os01g45570.1	100	3.78E-170	100
4	HD-ZIP	BGIOSGA002186-PA	LOC_Os03g43930.1	100	0	59
5	HD-ZIP	BGIOSGA004593-PA	LOC_Os01g57890.1	96	0	99
6	HD-ZIP	BGIOSGA005852-PA	LOC_Os02g45250.1	100	0	100
7	HD-ZIP	BGIOSGA006230-PA	LOC_Os02g35770.1	100	0	100
8	HD-ZIP	BGIOSGA007547-PA	LOC_Os02g05640.1	100	1.30E-173	100
9	HD-ZIP	BGIOSGA008720-PA	LOC_Os02g43330.1	100	0	99
10	HD-ZIP	BGIOSGA008976-PA	LOC_Os02g49700.1	100	0	99
11	HD-ZIP	BGIOSGA011276-PA	LOC_Os03g10210.1	100	3.79E-177	99
12	HD-ZIP	BGIOSGA011366-PA	LOC_Os03g07450.1	100	0	92
13	HD-ZIP	BGIOSGA011687-PA	LOC_Os03g01890.1	100	0	98
14	HD-ZIP	BGIOSGA012017-PA	LOC_Os03g08960.1	100	0	99
15	HD-ZIP	BGIOSGA012175-PA	LOC_Os03g12860.1	100	0	100
16	HD-ZIP	BGIOSGA013211-PA	LOC_Os03g43930.1	100	0	100
17	HD-ZIP	BGIOSGA014304-PA	LOC_Os04g53540.1	100	0	100
18	HD-ZIP	BGIOSGA014527-PA	LOC_Os04g48070.1	98	0	96
19	HD-ZIP	BGIOSGA014600-PA	LOC_Os04g46350.1	100	0	100
20	HD-ZIP	BGIOSGA016852-PA	LOC_Os04g45810.1	100	0	100
21	HD-ZIP	BGIOSGA020420-PA	LOC_Os01g45570.1	78	8.61E-33	43
22	HD-ZIP	BGIOSGA020603-PA	LOC_Os06g48290.1	100	0	99
23	HD-ZIP	BGIOSGA021700-PA	LOC_Os06g10600.1	88	0	96
24	HD-ZIP	BGIOSGA022270-PA	LOC_Os06g04850.1	100	0	100
25	HD-ZIP	BGIOSGA022272-PA	LOC_Os06g04870.1	100	0	100
26	HD-ZIP	BGIOSGA026014-PA	LOC_Os07g39320.1	100	8.32E-180	100
27	HD-ZIP	BGIOSGA026788-PA	LOC_Os08g37580.1	100	0	100
28	HD-ZIP	BGIOSGA026851-PA	LOC_Os08g36220.1	100	0	99
29	HD-ZIP	BGIOSGA026973-PA	LOC_Os08g32085.1	100	0	100
30	HD-ZIP	BGIOSGA027698-PA	LOC_Os08g04190.1	95	0	100
31	HD-ZIP	BGIOSGA028102-PA	LOC_Os08g08820.1	100	0	97
32	HD-ZIP	BGIOSGA028396-PA	LOC_Os08g19590.1	100	0	99
33	HD-ZIP	BGIOSGA029405-PA	LOC_Os09g35760.1	100	0	91
34	HD-ZIP	BGIOSGA029665-PA	LOC_Os09g27450.1	99	0	99
35	HD-ZIP	BGIOSGA029853-PA	LOC_Os09g21180.1	100	0	99
36	HD-ZIP	BGIOSGA030941-PA	LOC_Os09g29460.1	100	0	100
37	HD-ZIP	BGIOSGA031343-PA	LOC_Os10g42490.1	100	0	100
38	HD-ZIP	BGIOSGA032409-PA	LOC_Os10g01470.1	100	0	100
39	HD-ZIP	BGIOSGA032818-PA	LOC_Os10g23090.1	100	0	85
40	HD-ZIP	BGIOSGA032882-PA	LOC_Os10g26500.1	100	0	100
41	HD-ZIP	BGIOSGA033144-PA	LOC_Os10g33960.1	100	0	96
42	HD-ZIP	BGIOSGA033443-PA	LOC_Os10g41230.1	100	0	100
43	HD-ZIP	BGIOSGA035845-PA	LOC_Os12g41860.1	100	0	100
1	TALE	BGIOSGA000492-PA	LOC_Os01g62920.1	100	0	100
2	TALE	BGIOSGA003338-PA	LOC_Os01g19694.1	100	1.57E-82	100
3	TALE	BGIOSGA007823-PA	LOC_Os02g13310.1	100	0	100
4	TALE	BGIOSGA009878-PA	LOC_Os03g51710.1	100	0	91
5	TALE	BGIOSGA009880-PA	LOC_Os03g51690.1	100	0	87
6	TALE	BGIOSGA010051-PA	LOC_Os03g47740.1	100	0	100
7	TALE	BGIOSGA010052-PA	LOC_Os03g47730.1	100	0	98
8	TALE	BGIOSGA010071-PA	LOC_Os03g47036.1	39	2.61E-99	99
9	TALE	BGIOSGA010073-PA	LOC_Os03g47016.1	46	1.03E-115	100
10	TALE	BGIOSGA011390-PA	LOC_Os03g06930.1	100	0	100
11	TALE	BGIOSGA011746-PA	LOC_Os03g03164.1	100	0	100
12	TALE	BGIOSGA011748-PA	LOC_Os03g03260.1	100	0	100
13	TALE	BGIOSGA013510-PA	LOC_Os03g52239.1	100	0	88
14	TALE	BGIOSGA013637-PA	LOC_Os03g56110.1	63	0	96
15	TALE	BGIOSGA013638-PA	LOC_Os03g56110.1	100	0	85
16	TALE	BGIOSGA018883-PA	LOC_Os05g03884.1	100	0	100
17	TALE	BGIOSGA019968-PA	LOC_Os05g38120.1	100	0	95
18	TALE	BGIOSGA020786-PA	LOC_Os06g43860.1	100	0	100
19	TALE	BGIOSGA021033-PA	LOC_Os06g36680.1	88	0	100
20	TALE	BGIOSGA022158-PA	LOC_Os06g01934.1	100	0	99
21	TALE	BGIOSGA025091-PA	LOC_Os07g03770.1	100	0	100
22	TALE	BGIOSGA028399-PA	LOC_Os08g19650.1	91	5.33E-148	100
23	TALE	BGIOSGA031494-PA	LOC_Os10g39030.1	100	0	99
24	TALE	BGIOSGA034459-PA	LOC_Os11g06020.1	100	0	100
25	TALE	BGIOSGA035766-PA	LOC_Os12g43950.1	100	0	100
26	TALE	BGIOSGA036588-PA	LOC_Os11g06020.1	68	7.03E-85	79
27	TALE	BGIOSGA036589-PA	LOC_Os11g06020.1	100	0	59
1	WOX	BGIOSGA000469-PA	LOC_Os01g63510.1	100	3.76E-154	100
2	WOX	BGIOSGA000516-PA	LOC_Os01g62310.1	100	0	100
3	WOX	BGIOSGA001059-PA	LOC_Os01g47710.1	100	0	100
4	WOX	BGIOSGA004695-PA	LOC_Os01g60270.1	100	0	100
5	WOX	BGIOSGA010824-PA	LOC_Os03g20910.1	99	0	100
6	WOX	BGIOSGA014161-PA	LOC_Os04g56780.1	100	0	97
7	WOX	BGIOSGA014217-PA	LOC_Os04g55590.1	100	0	100
8	WOX	BGIOSGA019069-PA	LOC_Os05g02730.1	100	0	97
9	WOX	BGIOSGA020366-PA	LOC_Os05g48990.1	100	0	100
10	WOX	BGIOSGA026413-PA	LOC_Os07g48560.1	100	0	100
11	WOX	BGIOSGA027391-PA	LOC_Os08g14400.1	100	0	99
12	WOX	BGIOSGA036831-PA	LOC_Os12g01120.1	100	2.53E-154	100
1	ZF-HD	BGIOSGA001155-PA	LOC_Os01g44430.1	99	0	100
2	ZF-HD	BGIOSGA005769-PA	LOC_Os02g47770.1	87	9.14E-142	99
3	ZF-HD	BGIOSGA013464-PA	LOC_Os03g50920.1	100	5.07E-174	100
4	ZF-HD	BGIOSGA015067-PA	LOC_Os04g35500.1	61	2.45E-43	98
5	ZF-HD	BGIOSGA015069-PA	LOC_Os04g35500.1	53	5.95E-105	100
6	ZF-HD	BGIOSGA020427-PA	LOC_Os05g50310.1	100	0	99
7	ZF-HD	BGIOSGA026798-PA	LOC_Os08g37400.1	100	0	100
8	ZF-HD	BGIOSGA026924-PA	LOC_Os08g34010.1	81	0	98
9	ZF-HD	BGIOSGA028731-PA	LOC_Os09g29130.1	54	1.41E-23	59
10	ZF-HD	BGIOSGA029617-PA	LOC_Os09g29130.1	100	0	99
11	ZF-HD	BGIOSGA029765-PA	LOC_Os09g24820.1	88	2.56E-134	99
12	ZF-HD	BGIOSGA030752-PA	LOC_Os09g24810.1	100	3.75E-80	98
13	ZF-HD	BGIOSGA034224-PA	LOC_Os11g13930.1	89	2.96E-130	79
14	ZF-HD	BGIOSGA034718-PA	LOC_Os11g03420.1	100	2.02E-75	100
15	ZF-HD	BGIOSGA036923-PA	LOC_Os12g03110.1	100	9.78E-76	100

### 2.4 Putative *cis*-regulatory element analysis

#### 2.4.1 Retrieval of predicted *HB* gene promoters in rice and chromosomal localization of genes

For this analysis, the genome of *Oryza sativa* subsp. *japonica* var. Nipponbare (version 7.0) was used in FASTA (sequence) and GFF3 formats. The six predicted *HB* gene subfamilies of *O. sativa* subsp. *japonica* (*i.e.*, HB-PHD, HD-ZIP, TALE, WOX, ZF-HD, and HB-OTHERS) were analyzed. Gene annotations were extracted from the GFF3 file and subsequently filtered to retain those corresponding to each HB subfamily. Promoter regions were operationally defined as the 1, 000-bp genomic sequence immediately upstream of the annotated gene boundary in the transcriptional orientation. For genes on the positive strand, the interval extended from 1, 000 bp upstream to one base before the annotated gene start; for genes on the negative strand, the corresponding interval was extracted downstream in genomic coordinates and reverse-complemented. Promoter sequences were extracted with BEDTools v2.31.0 (Quinlan, 2014) from the Nipponbare MSU v7.0 genome. Chromosomal positions of validated *HB* loci were visualized separately using TBtools-II v2.326 (Chen et al., 2023).

#### 2.4.2 Putative *cis*-regulatory elements

Promoter sequences of all predicted *HB* genes were submitted to PlantCARE (http://bioinformatics.psb.ugent.be/webtools/plantcare/html/) (Lescot et al., 2002) for prediction of putative *cis*-regulatory elements. All returned annotations were retained in the complete dataset. Elements were subsequently grouped into stress-, hormone-, and development-associated categories according to PlantCARE annotations. Both the total number of motif occurrences and the proportion of promoters containing each motif were calculated. The complete unfiltered annotation is provided in **Supplementary Material SM1**.

#### 2.4.3 *De novo* motif discovery

*De novo* motif discovery was performed separately for the promoter set of each HB subfamily using MEME v5.5.9 (Bailey et al., 2015). Motifs were searched on both DNA strands under the ANR (Any Number of Repetitions) occurrence model, with a maximum of three motifs and motif widths ranging from six to 50 bp. A first-order Markov background model derived from the corresponding promoter sequences was used. Motifs with an *E*-value below 0.05 were retained. Similarity to known plant transcription-factor binding profiles was assessed using Tomtom v5.5 (Gupta et al., 2007) against the JASPAR CORE 2026 Plants collection (https://jaspar.elixir.no/collection/core/) (Ovek-Baydar et al., 2026). Matches with a false-discovery-rate-adjusted q-value below 0.05 were considered significant. Low-complexity motifs and matches without significant similarity were reported but not assigned to specific transcription-factor families.

### 2.5 Analysis of gene expression of candidate *HB* genes

One candidate gene from each predicted *HB* subfamily was selected *a priori* for exploratory expression profiling based on the diversity of predicted stress- and hormone-associated promoter motifs. These genes were not assumed to represent the complete expression behavior of their respective subfamilies, but rather to have a first overview regarding their expression patterns under different developmental stages, tissues and stress conditions Publicly available rice expression data were retrieved through eFP-Plant platform (https://bar.utoronto.ca/eplant_rice/). The original experimental datasets are summarized in **Supplementary Material SM2** and **SM3**. Expression values were standardized separately for each gene using $z=\left(x-\bar{x}\right)/s$ (Abdi and Williams, 2010). Positive and negative *z*-scores indicate values above or below the mean expression of that gene across the samples displayed, respectively, and were not interpreted as statistically significant differential expression unless supported by the original dataset.

All protein and promoter sequences, domain-validation results, BLASTp outputs, multiple-sequence alignments, phylogenetic trees, motif predictions, and expression matrices used in this study are provided as **Supplementary Material** (**SM1**–**SM8**).

## 3 Results

### 3.1 Distribution of the predicted *Homeobox* gene family in species and subspecies of the genus *Oryza*

According to sequence data from the PlantTFDB database, we manually verified these sequences and retrieved a total of 112 candidate *HB* genes in *Oryza sativa* subsp. *indica* and 156 in *O. sativa* subsp. *japonica*. In addition, the following numbers of candidate *HB* genes were retrieved in other *Oryza* species: 116 in *O. barthii*, 94 in *O. brachyantha*, 95 in *O. glaberrima*, 124 in *O. glumaepatula*, 66 in *O. longistaminata*, 121 in *O. meridionalis*, 131 in *O. nivara*, 132 in *O. punctata*, and 125 in *O. rufipogon* (**Figure 1**).

**Figure 1:**
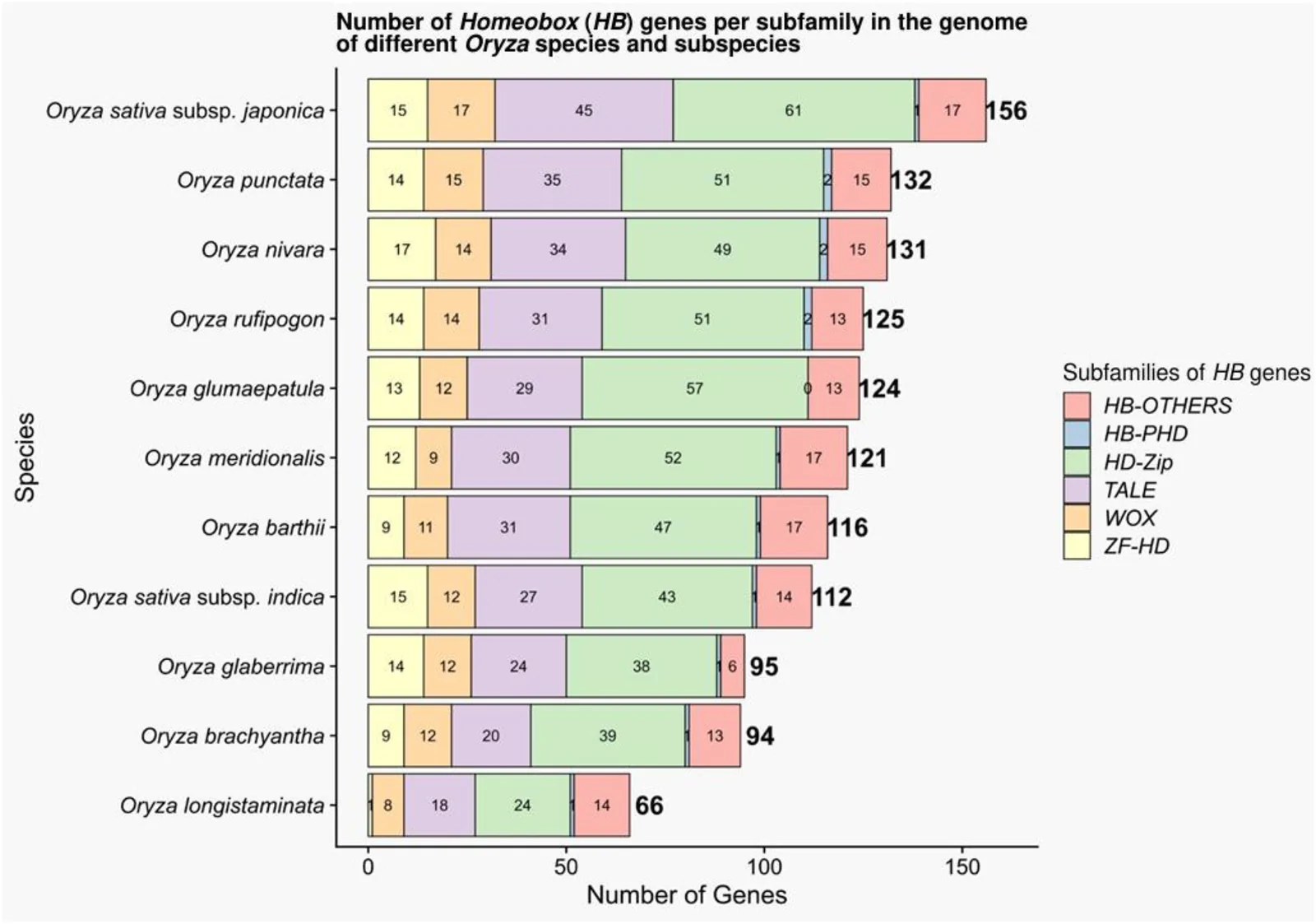
Predicted number of *Homeobox* (*HB*) genes per subfamily in the genome of different *Oryza* species and subspecies, in accordance to the Plant Transcription Factor Database (http://planttfdb.gao-lab.org/) (Jin et al., 2017). *HB-PHB*, Homeobox-plant homeodomain; *HB-OTHERS*, include *HB* genes different from the rest of the subfamilies; *HD-Zip*, Homeodomain-leucine zipper motif; *TALE*, three-amino-acid-loop-extension; *WOX*, WUSCHEL homeobox-containing protein; *ZF-HD*, zinc-finger homeodomain protein.

### 3.2 Identification of best-hit homologs between *O. sativa* subspecies

The results showed a high degree of conservation among HB proteins in *Oryza sativa* subsp. *indica* and *O. sativa* subsp. *japonica*. Overall, the 112 *O. sativa* subsp. *indica* sequences exhibited an average coverage of 91% and a mean identity of 94% compared to *O. sativa* subsp. *japonica*. The HB-PHD subfamily reached the highest identity value (100%), whereas the HD-ZIP, TALE, WOX, and ZF-HD subfamilies displayed identity values equal to or greater than 95%. These findings may indicate strong evolutionary conservation between the two subspecies. In contrast, the HB-OTHERS subfamily showed the lowest identity (82%), suggesting lower evolutionary conservation within this group (**Table 1**).

### 3.3 Phylogenetic analysis of predicted HB transcription factors in rice

The phylogenetic tree was constructed using the 223 HB transcription factor peptide sequences based on the maximum likelihood (ML) method (**Figure 2**). The analysis revealed a well-supported and reliable topology. According to the previously reported classification for this family (Jin et al., 2017; Tian et al., 2020) and the inferred topology, the proteins were grouped into six main clades: HB-PHD, TALE, WOX, ZF-HD, HD-ZIP, and HB-OTHERS. The HB-PHD, TALE, WOX, and ZF-HD subfamilies formed more consistent clusters, whereas HD-ZIP and HB-OTHERS were distributed across different clades, suggesting greater evolutionary diversity within these groups.

**Figure 2:**
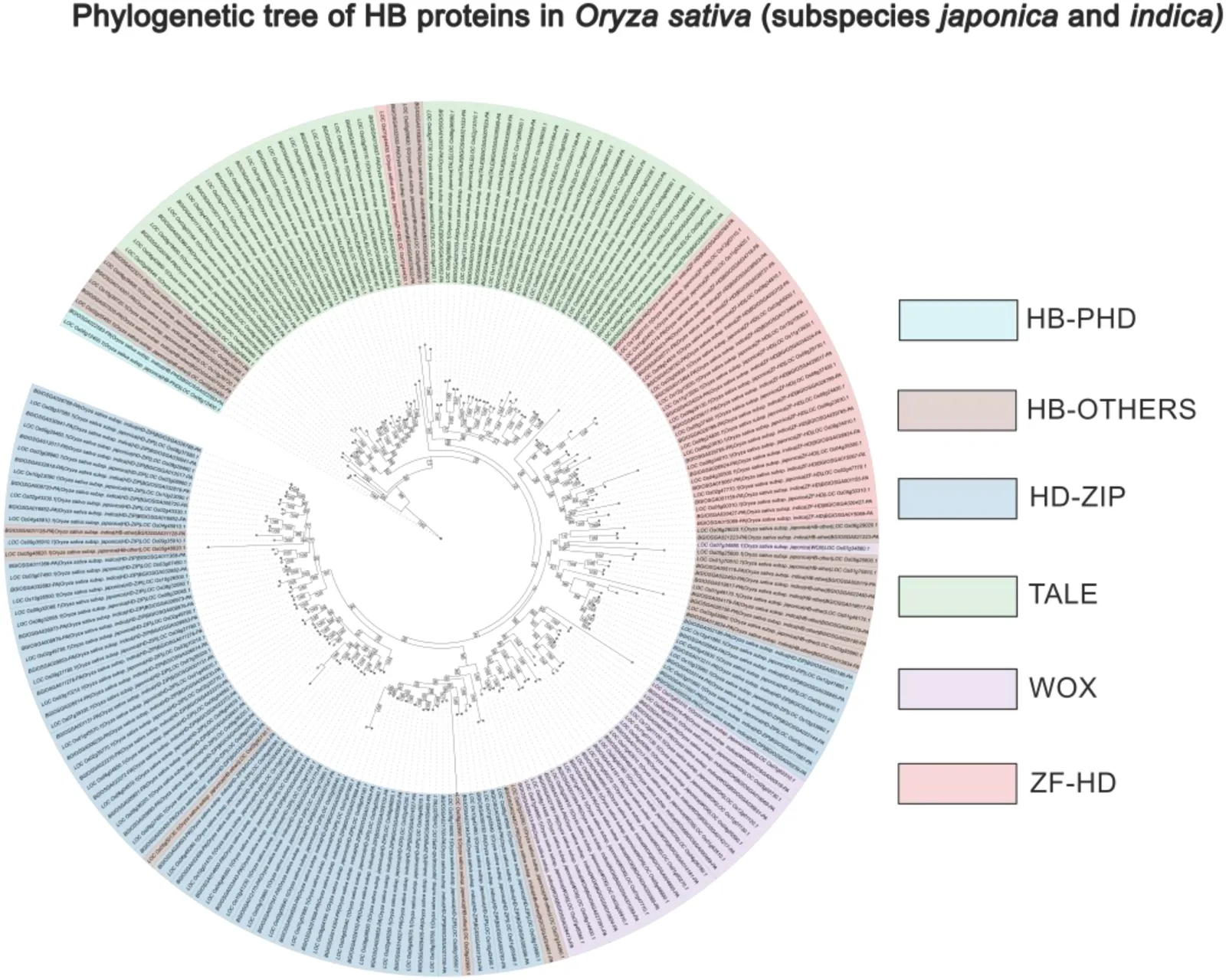
Phylogenetic tree of predicted HB proteins in *Oryza sativa* subsp. *indica* and subsp. *japonica*, constructed using 223 protein sequences from six HB subfamilies based on the maximum likelihood (ML) method with 1, 000 bootstrap replicates.

Further analysis using InterProScan revealed a high degree of concordance between the detected conserved domains and the phylogenetic classification of the HB proteins. HD-ZIP proteins primarily featured homeodomains accompanied by leucine zipper-associated regions and, in certain members, START domains. TALE proteins displayed domains characteristic of this family—including KN Homeobox, KNOX1, KNOX2, and ELK—while WOX proteins consistently showed annotations related to homeodomains. The 30 ZF-HD proteins exhibited the characteristic ZF-HD dimerization domain, and HB-PHD proteins displayed both homeobox and PHD-finger domains. The HB-others group exhibited a more heterogeneous domain architecture. Overall, these results provide independent evidence that strongly supports the classification of the analyzed proteins (**Supplementary Material SM4–SM6**).

### 3.4 Chromosomal localization of *HB* genes

The chromosomal distribution of *HB* gene subfamily members spanned all 12 chromosomes of the rice genome. Chromosome 3 contained the highest number, with 21 genes, followed by chromosomes 8, 1, and 6, with 12, 11, and 11 genes, respectively. Chromosome 2 harbored nine genes, whereas chromosomes 10, 5, and 9 each contained eight genes. Chromosome 4 included seven genes, chromosomes 12 and 7 each had five genes, and chromosome 11 contained four genes. Notably, the TALE subfamily was overrepresented on chromosome 3, with 12 of its 26 genes (46% of this subfamily) (**Figure 3**).

**Figure 3:**
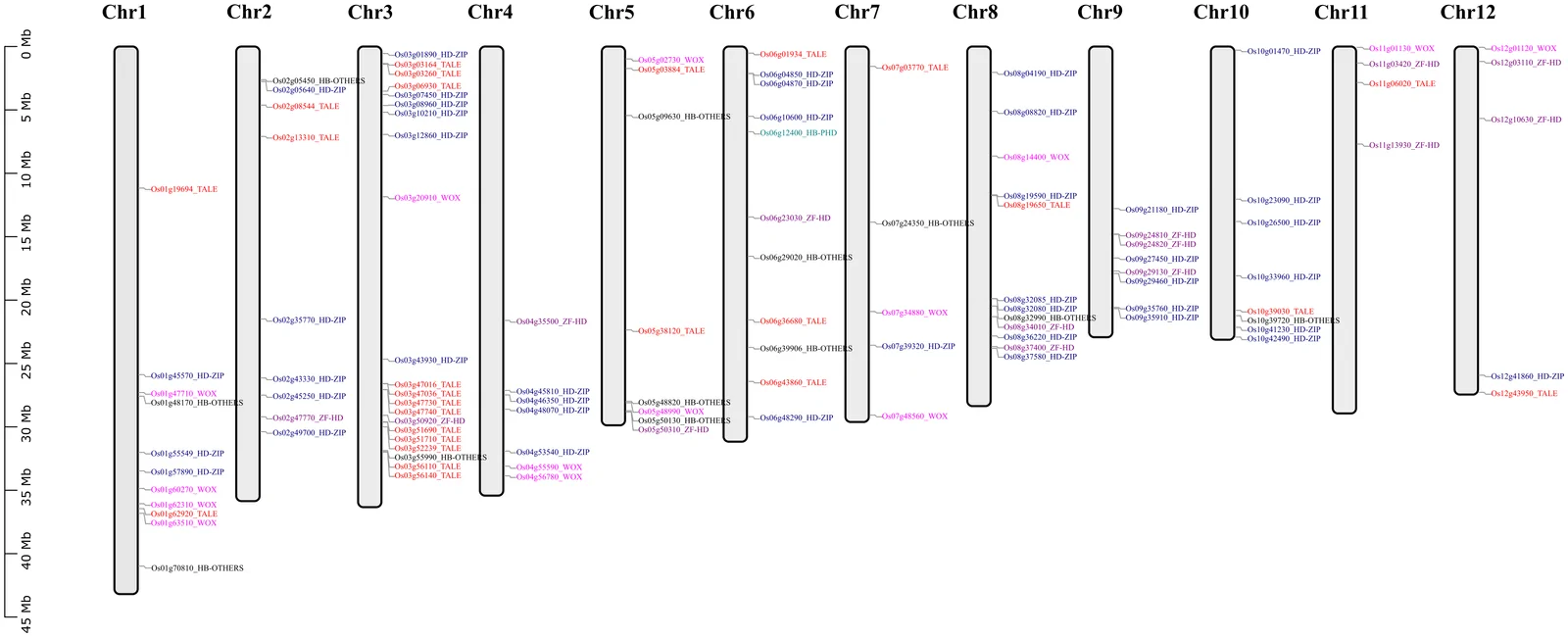
Chromosomal distribution of *HB* gene subfamilies in the *Oryza sativa* subsp. *japonica* genome.

### 3.5 Predicted *cis*-regulatory elements in promoters

The list of predicted motifs and their frequencies identified in the promoter regions of the analyzed *HB* genes is displayed in **Table 2**. The most frequent motif was ABRE (199 occurrences), followed by the CGTCA-motif and TGACG-motif, each with 102 occurrences. The least represented motifs were AuxRE and DRE, each detected once.

**Table 2:** List of putative motifs and their frequencies predicted in the promoter regions of genes from the six *HB* subfamilies in rice.

Number	Putative motifs	Predicted functions
199	ABRE	*cis*-acting element involved in the abscisic acid responsiveness
102	CGTCA-motif	*cis*-acting regulatory element involved in the MeJA-responsiveness
102	TGACG-motif	*cis*-acting regulatory element involved in the MeJA-responsiveness
98	ARE	*cis*-acting regulatory element essential for the anaerobic induction
42	MBS	MYB binding site involved in drought-inducibility
27	GC-motif	enhancer-like element involved in anoxic specific inducibility
26	LTR	*cis*-acting element involved in low-temperature responsiveness
26	TGA-element	auxin-responsive element
19	TC-rich repeats	*cis*-acting element involved in defense and stress responsiveness
17	P-box	gibberellin-responsive element
12	GARE-motif	gibberellin-responsive element
11	AuxRR-core	*cis*-acting regulatory element involved in auxin responsiveness
5	TATC-box	*cis*-acting element involved in gibberellin-responsiveness
1	AuxRE	part of an auxin-responsive element
1	DRE	*cis*-acting element involved in dehydration, low-temperature, and salt stress

All six *HB* gene subfamilies (*i.e.*, HB-PHD, HB-OTHERS, HD-ZIP, TALE, WOX, and ZF-HD) predicted in the rice genome were detailed analyzed.

After filtering *cis*-regulatory elements with known functions, 688 motifs were predicted to be distributed across 15 different types associated with abiotic stress and phytohormone responses. Four motifs—ABRE (28.9%), CGTCA-motif (14.8%), TGACG-motif (14.8%), and ARE (14.2%)—accounted for 73% of the total, whereas the remaining 27% corresponded to the other 11 identified types. The ABRE motif showed the highest number of occurrences in the HD-ZIP, TALE, HB-OTHERS, ZF-HD, and WOX families, with 99, 35, 26, 21, and 16 occurrences, respectively. In contrast, the TGA-element showed its highest representation in the HB-PHD subfamily, with three occurrences (**Figure 4**).

**Figure 4:**
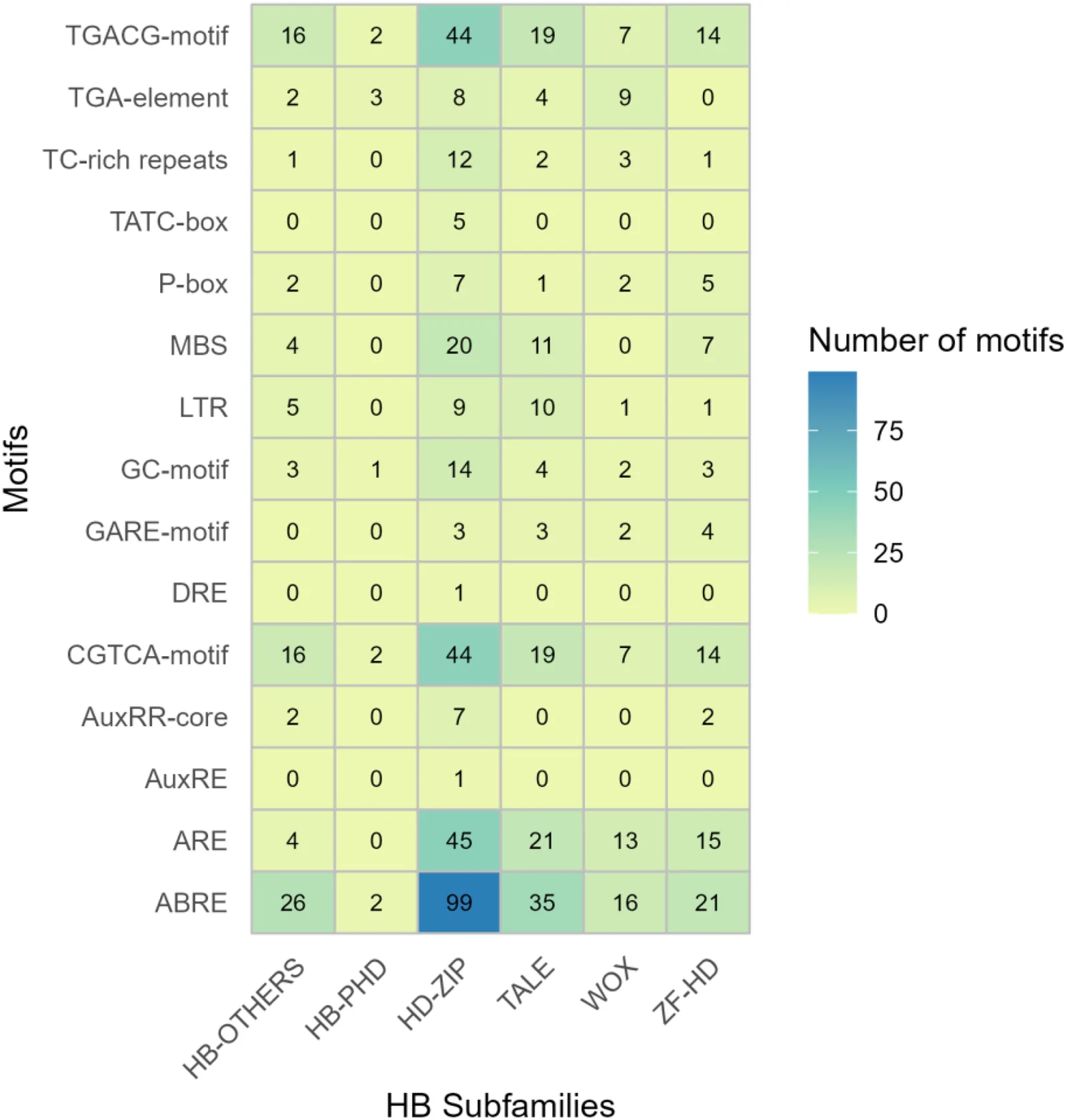
Heatmap representation of putative motif occurrence counts in the promoter regions of selected representative genes from different *HB* subfamilies. The color gradient ranges from yellow (lowest values) to green (intermediate values) and blue (highest occurrence counts). Motifs correspond to those listed in **Table 2**.

### 3.6 *De novo* motif discovery in promoters

In addition to identifying known motifs in promoters, *de novo* motifs were identified for each *HB* gene subfamily in this study. Within the *HB-OTHERS* subfamily, two putative motifs were detected (**Figure 5**): 1) consensus sequence CCYCCYCHKCSKCCGCCBCCBCCCYCKCC, with an *E*-value of 6.5e^−50^, present at 47 sites and associated with motifs previously reported in upstream regions of genes encoding AP2/EREBP (APETALA2/ethylene-responsive element binding protein) class proteins (Riechmann and Meyerowitz, 1998); and 2) consensus sequence DAAAWDWRRAWAAAAATRRA, with an *E*-value of 1.1e^−10^, present at 55 sites and associated with motifs previously identified in promoters of genes belonging to the Other C4 zinc-finger-type factor class (Duan et al., 2026).

**Figure 5:**
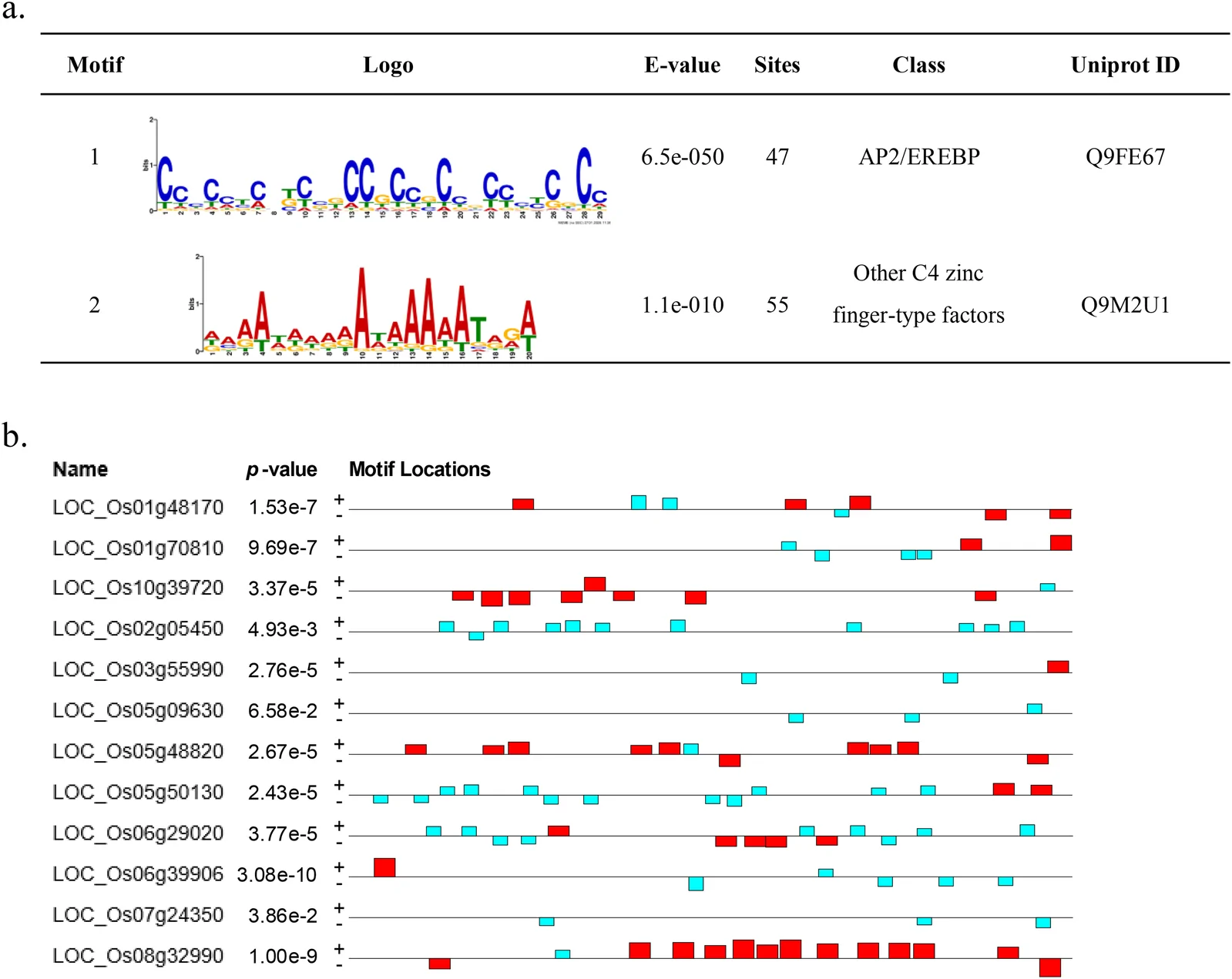
Putative motif analysis in the promoter region of *HB-OTHERS* genes in rice. **(a)** Values and references for each motif. **(b)** Motifs mapped onto each sequence; the width, position, and height of each box represent motif length, location within the promoter sequence, and statistical significance (*p*-value), respectively. Motifs are color-coded as follows: red for motif 1 and light blue for motif 2.

Similarly, at least three putative motifs were identified in the HD-ZIP subfamily (**Figure 6**): 1) consensus sequence CSSBCBSCBCCTCYCBCHCCCYCBCBCHC, with an *E*-value of 5.5e^−87^, present at 112 sites and associated with motifs previously reported in promoters of genes belonging to the BBR/BPC (Barley B Recombinant/Basic PentaCysteine) class (Sahu et al., 2023); 2) consensus sequence TRGAKTAATTAGGCTYAAWARATTYGTCTCGYRA, with an *E*-value of 1.5e^−40^, present at nine sites and associated with known motifs of the homeodomain factor class (Banerjee-Basu and Baxevanis, 2001); and 3) consensus sequence AAAAAWKAAAAAAAAAAHWAAWWATADAW, with an *E*-value of 3.1e^−28^, present at 85 sites and associated with motifs of the Other C4 zinc-finger-type factor class (Duan et al., 2026).

**Figure 6:**
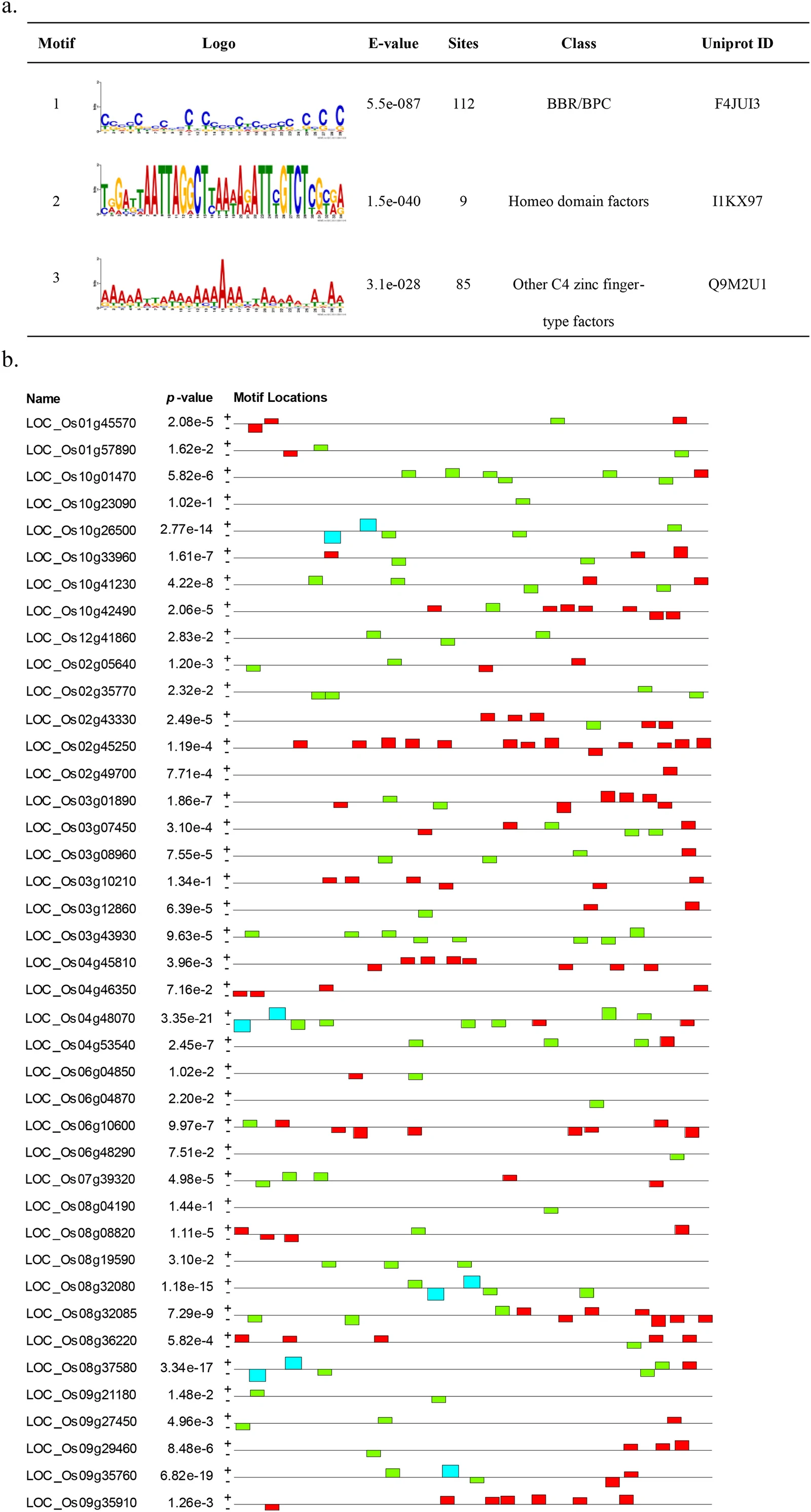
Putative motif analysis in the promoter region of *HD-ZIP* genes in rice. **(a)** Values and references for each motif. **(b)** Motifs mapped onto each sequence; the width, position, and height of each box represent motif length, location within the promoter sequence, and statistical significance (*p*-value), respectively. Motifs are color-coded as follows: red for motif 1, light blue for motif 2, and green for motif 3.

In the *TALE* subfamily, at least three putative motifs were identified: 1) WKGARAKRGAGRRAGARRRRR, with an *E*-value of 4.6e^−53^, present at 99 sites and associated with known motifs of the C2H2 zinc-finger like factor class; 2) WWTWTAWRTWTKWWTWAAWWTWTWKAWAA, with an *E*-value of 3.5e^−9^, present at 30 sites and associated with motifs of the CPP (cell-penetrating peptide) class; and 3) GCGGBGGNMRKNMCGGGGGGWBMMSDKYSGTSWGVYCGKSGGYSGSGAC, with an *E*-value of 4.4e^−6^, present at six sites and associated with motifs of the B3 class (**Figure 7**).

**Figure 7:**
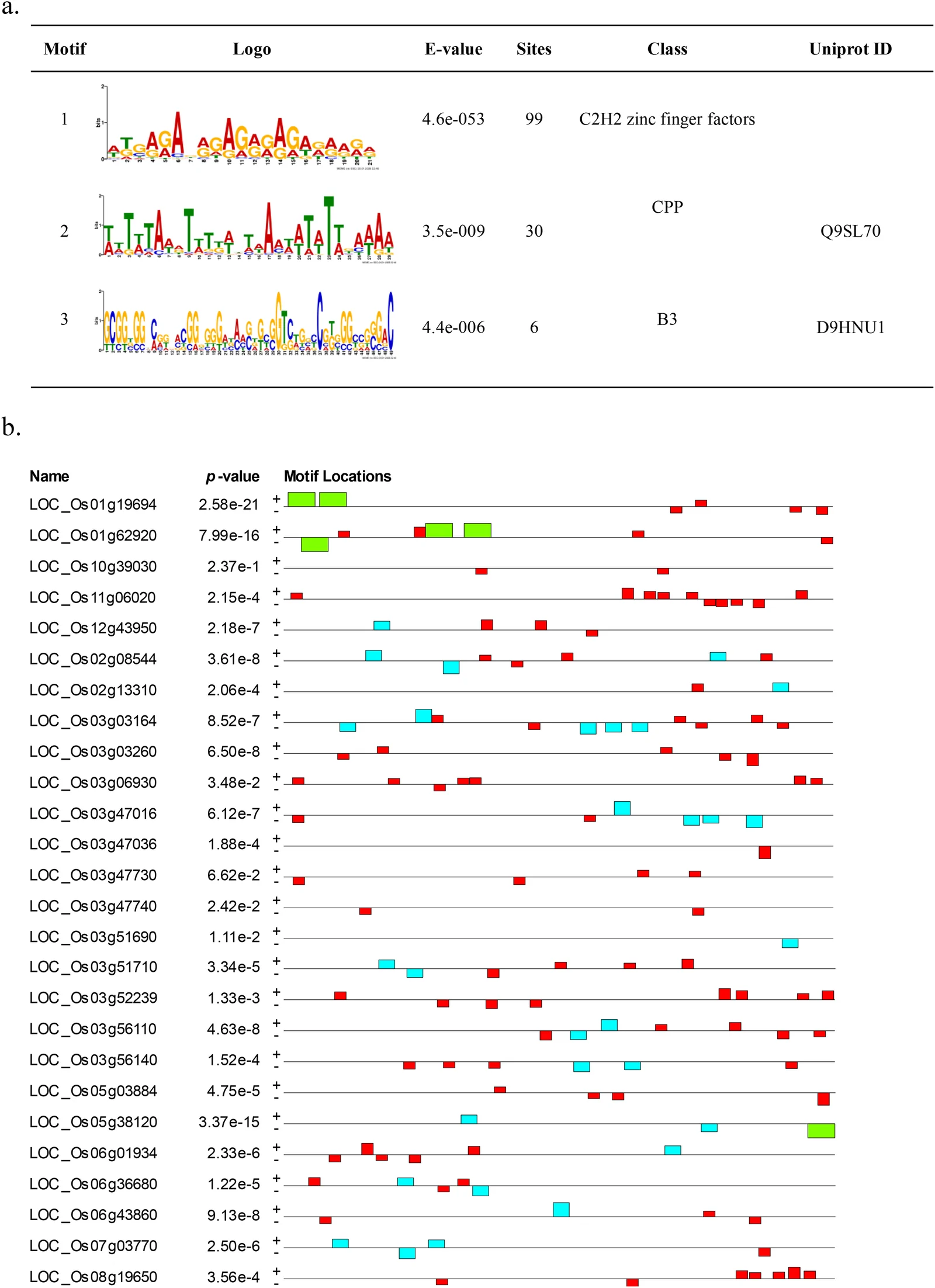
Putative motif analysis in the promoter region of *TALE* genes in rice. **(a)** Values and references for each motif. **(b)** Motifs mapped onto each sequence; the width, position, and height of each box represent motif length, location within the promoter sequence, and statistical significance (*p*-value), respectively. Motifs are color-coded as follows: red for motif 1, light blue for motif 2, and green for motif 3.

In the *WOX* subfamily, at least three putative motifs were identified (**Figure 8**): 1) CKCCYBCMTCHYCTCCHYCGCCCTCMYCC, with an *E*-value of 5.1e^−34^, present at 33 sites and associated with motifs of the BBR/BPC (Barley B Recombinant/Basic Pentacysteine) class (Santi et al., 2003; Kooiker et al., 2005); 2) WVARWGKTTCARYTRVTDTHYWSATMRTGTTTCATDTTTTT, with an *E*-value of 1.1e^−30^, present at 16 sites and associated with motifs of the Other C4 zinc-finger-type factor class (Duan et al., 2026); and 3) ATRSATHASYAGCWHWBBAKMTAGCKHKACYAGCKVSADYGACWCGAT, with an *E*-value of 3.5e^−9^, present at five sites and associated with motifs of the C3H(C), C2HC zinc-finger-like factor class (Duan et al., 2026).

**Figure 8:**
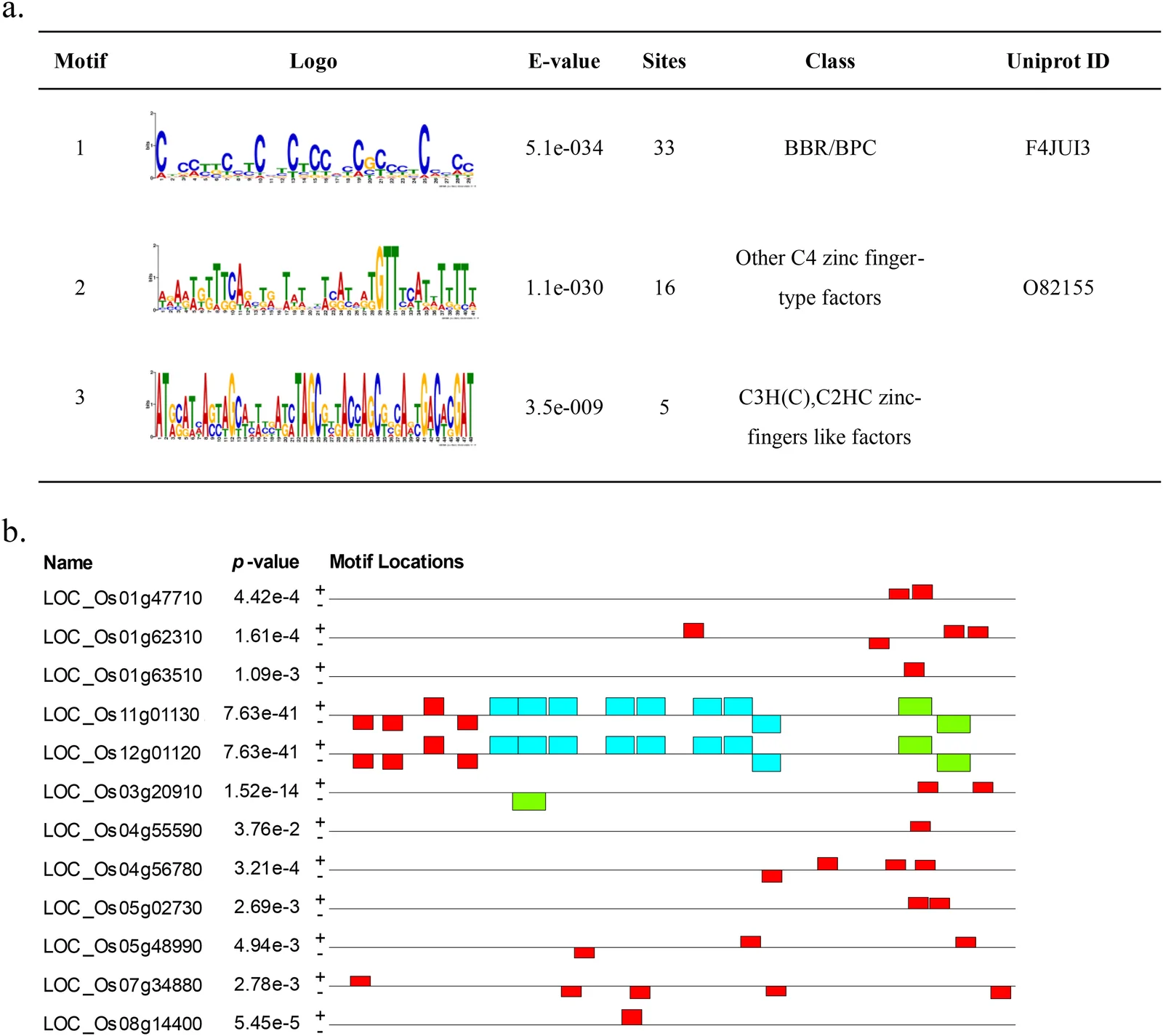
Putative motif analysis in the promoter region of *WOX* genes in rice. **(a)** Values and references for each motif. **(b)** Motifs mapped onto each sequence; the width, position, and height of each box represent motif length, location within the promoter sequence, and statistical significance (*p*-value), respectively. Motifs are color-coded as follows: red for motif 1, light blue for motif 2, and green for motif 3.

Finally, at least three putative motifs were identified in the *ZF-HD* subfamily (**Figure 9**): 1) YCYCNCYSYCKCCKCCNCYCC, with an *E*-value of 3.2e^−41^, present at 33 sites and associated with motifs of the AP2/EREBP class (Riechmann and Meyerowitz, 1998); 2) MARMAAARVWAWNTHKWTTKHAARDDDAWWATAWDAHAAAAHAAGYAAMA, with an *E*-value of 2.8e^−17^, present at 16 sites and associated with motifs of the Other C4 zinc-finger-type factor class (Duan et al., 2026); and 3) AAAAANRWAAGADAAAAWRAA, with an *E*-value of 1.5e^−4^, present at five sites and associated with motifs of the Other C4 zinc-finger-type factor class (Duan et al., 2026).

**Figure 9:**
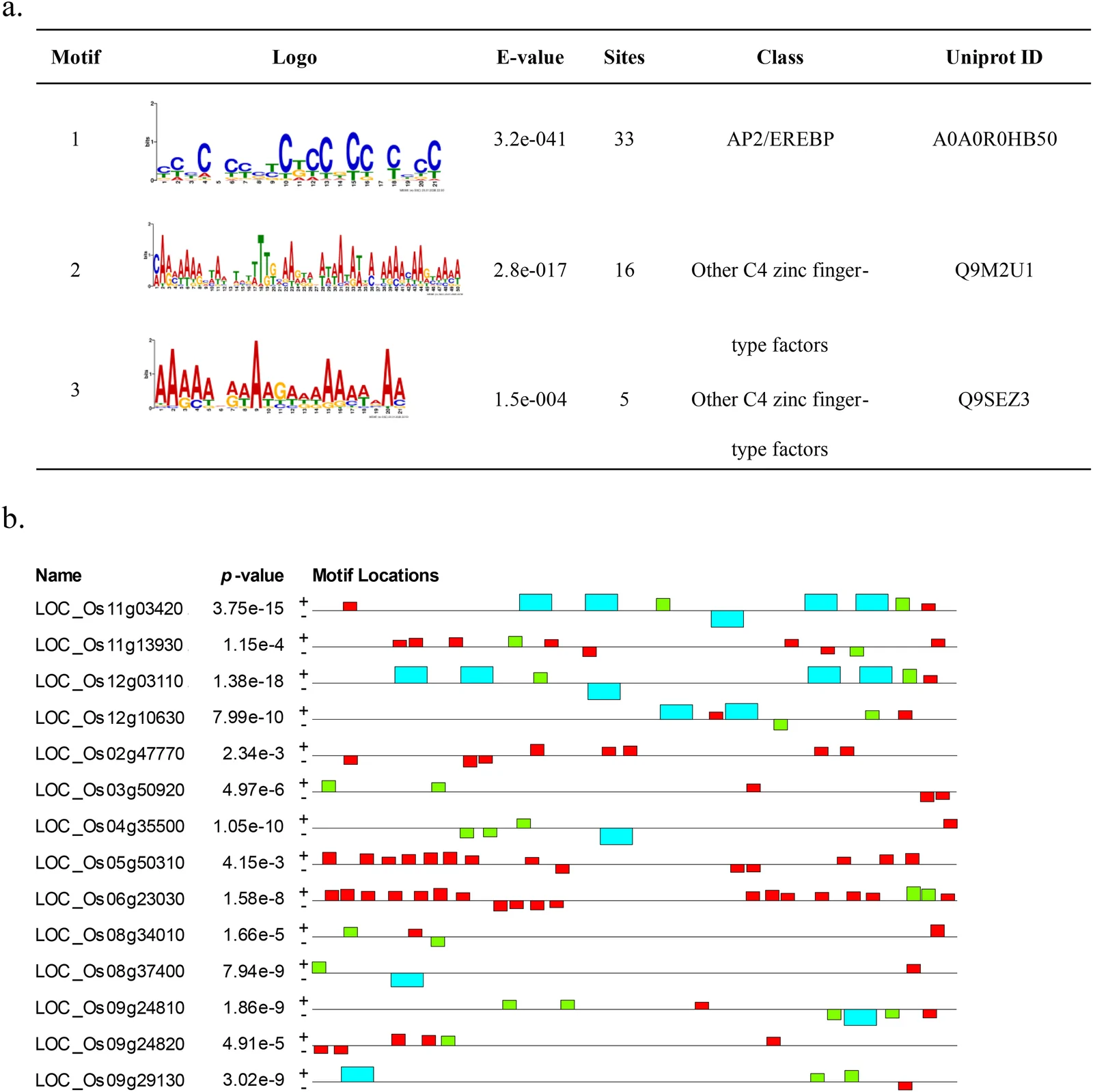
Putative motif analysis in the promoter region of *ZF-HD* genes in rice. **(a)** Values and references for each motif. **(b)** Motifs mapped onto each sequence; the width, position, and height of each box represent motif length, location within the promoter sequence, and statistical significance (*p*-value), respectively. Motifs are color-coded as follows: red for motif 1, light blue for motif 2, and green for motif 3.

### 3.7 Gene expression

For gene expression analysis, one candidate gene per predicted *HB* subfamily was selected *a priori* based on promoter motif diversity.

The *in-silico* gene expression results normalized by *z*-scores showed distinct patterns depending on the stress condition. A *z*-score close to 0 indicates that gene expression is near its mean level, whereas positive values (*z* > 0) denote expression above the mean (upregulation), and negative values (*z<* 0) imply expression below that mean (downregulation). The gene *LOC_Os11g03420* (ZF-HD) showed clear induction under cold (0.3) and drought (0.6) conditions but was strongly repressed by salinity (−0.9), suggesting a role in responses to low temperature and water deficit. In contrast, *LOC_Os06g12400* (HB-PHD) exhibited lower relative expression under cold (−1.2) and upregulation under salinity (1.0). Likewise, *LOC_Os03g03260* (TALE) was highly induced under salinity (1.2). Moreover, *LOC_Os05g48990* (WOX) displayed increased transcript abundance under drought conditions (0.5), whereas *LOC_Os03g12860* (HD-ZIP) showed moderate induction expression under salinity (0.4). Finally, *LOC_Os01g70810* (HB-OTHERS) displayed moderate positive standardized expression values under both drought and salinity (0.2). Overall, the studied *HB* genes in rice exhibited differential expression, with higher activity under drought and salinity and more moderate responses under cold stress, highlighting their potential role as key regulators of adaptation under adverse conditions (**Figure 10**).

**Figure 10:**
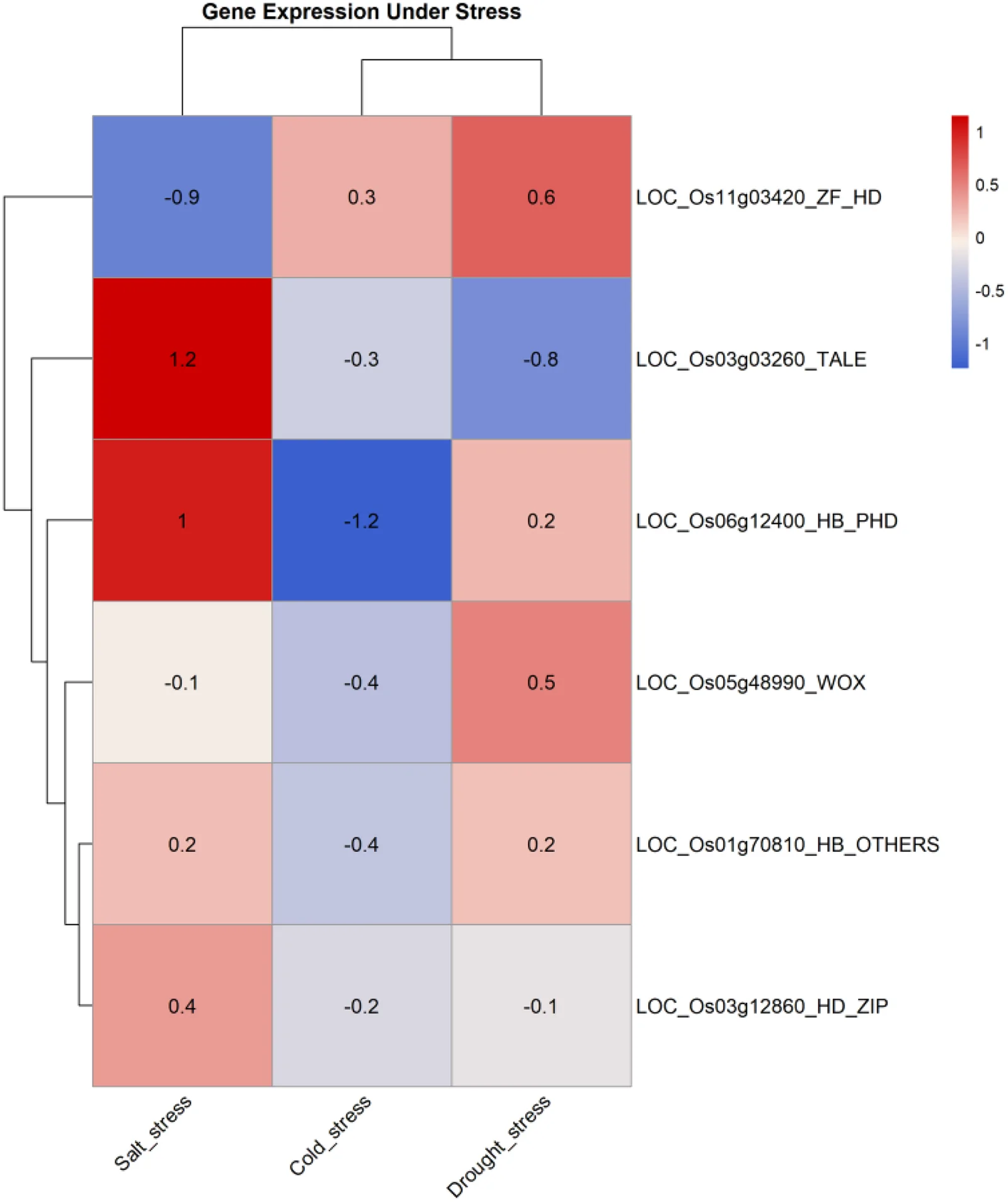
Heatmap of gene expression under different stress conditions (salinity, cold, and drought) for selected representative genes of each *HB* subfamily. Values in the heatmap correspond to *z*-score normalization, calculated by centering each gene distribution value to its mean and dividing by its standard deviation. Values close to or equal to 0 represent the average expression of each gene; positive values (*z* > 0) indicate expression above the mean standardized expression of that gene across the displayed samples; negative values (*z<* 0) indicate expression below that mean.

The normalized gene expression analysis revealed distinct patterns across different stages of rice development. In particular, *LOC_Os01g70810* (HB-OTHERS) showed elevated expression in SAM (1.6), Young Inflorescence (1.4), and Young Leaf (0.7), as well as in Inflorescence_P4 (0.5), suggesting a key role in young tissues and inflorescence development. *LOC_Os03g03260* (TALE) exhibited the highest induction in Mature Leaf (3.4) and moderate expression in Young Leaf (0.6), indicating that this gene may be important for leaf development and possibly involved in photosynthesis. *LOC_Os03g12860* (HD-ZIP) was highly expressed in Seed_S3 (1.0), Seed_S4 (1.1), Seed_S5 (0.5), Inflorescence_P5 (0.4), and Inflorescence_P6 (0.2), suggesting its possible involvement in seed development and late inflorescence stages. Similarly, *LOC_Os05g48990* (WOX) showed upregulation during seed and inflorescence development, specifically in Seed_S1 (0.8), Seed_S3 (0.8), Seed_S4 (1.2), Seed_S5 (1.0), Young Inflorescence (0.3), and Inflorescence_P5 (0.1). Likewise, *LOC_Os06g12400* (HB-PHD) exhibited high expression in Inflorescence_P2 and Inflorescence_P3 (1.9–0.3) and in seeds at Seed_S2 (0.3), Seed_S3 (0.6), Seed_S4 (0.4), and Seed_S5 (1.1), highlighting its role in both inflorescence and seed development. Finally, *LOC_Os11g03420* (ZF-HD) showed induced expression in Seed_S2 (0.5), Seed_S3 (1.3), Seed_S4 (1.9), and Seed_S5 (2.1), suggesting a prominent function in seed growth and maturation (**Figure 11**).

**Figure 11:**
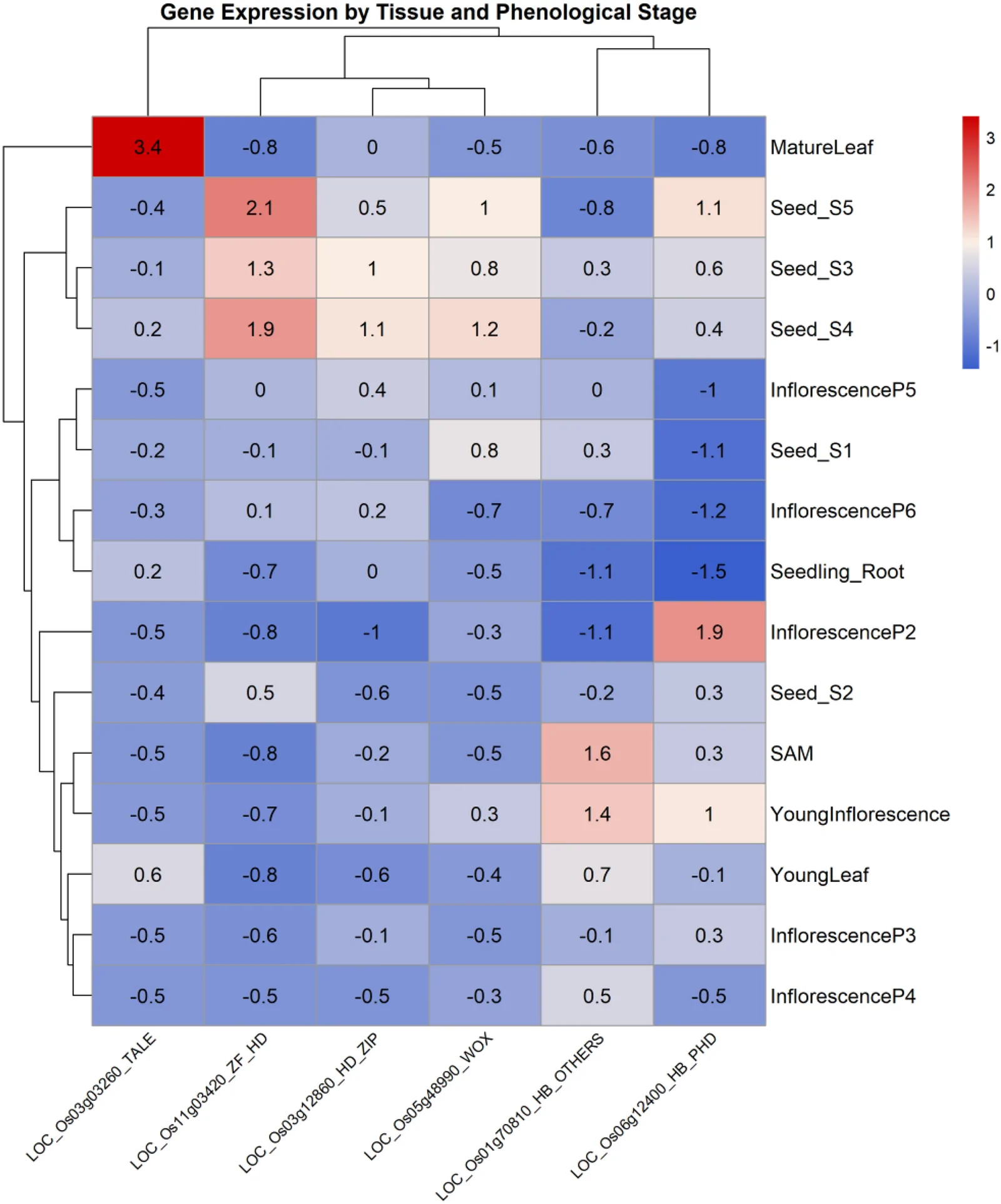
Heatmap of gene expression across different tissues and developmental stages of selected representative genes of each *HB* subfamily. Values represent *z*-scores, calculated by centering each gene distribution value to its mean and dividing by its standard deviation; Values close to or equal to 0 represent the average expression of each gene; positive values (*z* > 0) indicate expression above the mean standardized expression of that gene across the displayed samples; negative values (*z<* 0) indicate expression below that mean.

## 4 Discussion

In this study we were able to retrieve a total of 112 and 156 sequences annotated as HB transcription factors from PlantTFDB for *O. sativa* subsp. *indica* and subsp. *japonica*, respectively. These sequences showed 94% sequence identity, which represented the simple average of the identity percentages of the best hits obtained via BLASTp.

The comparative analysis of the distribution of the *Homeobox* (*HB*) gene family across different species of the genus *Oryza* revealed variability in gene number among subspecies, ranging from 66 in *Oryza longistaminata* to 156 in *Oryza sativa* subsp. *japonica* (**Figure 1**). This variability suggests that the *HB* gene family may have undergone lineage diversification over time. The higher number of genes in *O. sativa* subsp. *japonica* may reflect its potential evolution of adaptive features (Vaughan et al., 2008; Jain et al., 2008; Mukherjee et al., 2009). However, the variance in the number of retrieved *Homeobox* genes between the explored *Oryza* datasets cannot be definitively attributed to evolutionary expansion or adaptation. While biological variation and dynamic evolutionary events are plausible explanations, they remain speculative until technical and methodological variables are strictly controlled (Li and Jiong, 2024; Li et al., 2025b).

Another important observation was that the *HD-ZIP* subfamily contained the largest number of predicted genes among *HB* transcription factors, suggesting a prominent role in development and responses to stress conditions, thereby highlighting it as one of the most relevant subfamilies among this family of transcription factors (Agalou et al., 2008; Zhang et al., 2012; Li et al., 2022; Ahangarani-Farahani et al., 2025). Interestingly, the second most diverse subfamily was TALE [also referred to as KNOX (KNOTTED1-like homeobox) and BELL (BEL-like homeobox)]. TALE genes and proteins can be induced by abiotic adversities such as dehydration, cold, and NaCl stress (Xie et al., 2024; Guan et al., 2025). BEL-like homeobox transcription factors have been found to play crucial roles in plant development and environmental responses (Niu and Fu, 2022). In rice, the BELL-type homeobox transcription factor RLC3/OsBLH4 controls leaf rolling and drought tolerance via KNOX-BELL-lignin regulatory network (Qiao et al., 2026). Whether the retrieved *HB* genes reported in this study are indeed responsive to osmotic stress (dehydration, salinity or cold stress) remains to be elucidated.

Based on the results of the phylogenetic relationship (**Figure 2**) and chromosome localization analysis (**Figure 3**), we found that some *HB* genes located on the same chromosome were also clustered together in the phylogenetic tree. For example, most *TALE* genes located on chromosome 3 are clustered together. These results suggest that they may have evolved from gene duplication events and might be functionally redundant (Guan et al., 2025). This clustering indicates that gene duplication events might have played a role in the expansion and functional diversification of the *HB* genes in rice. Interestingly, the HB transcription factor family shows both conserved and more divergent clades, which could indicate the presence of processes of conservation and evolutionary diversification. The HB-PHD, TALE, WOX, and ZF-HD groups exhibit greater phylogenetic consistency, while HD-ZIP and HB-OTHERS show a more dispersed distribution in the tree. Overall, these results show that some groupings are more consistent than others within the analyzed groups, which is consistent with previous studies (Bhattacharjee et al., 2015; Viola and Gonzalez, 2016; Joo et al., 2018; Hou et al., 2021; Kanchan et al., 2025; Zhang et al., 2025a).

A considerable difference was observed between the number of *HB* genes identified in *O. sativa* subsp. *indica* (112) and *O. sativa* subsp. *japonica* (156). Further analysis of conserved domains showed that the proteins included in the phylogenetic analysis generally exhibit domain architectures consistent with their respective HB groups; this reduces the likelihood that the observed difference could be broadly attributed to the inclusion of proteins lacking characteristics typical of HB families. However, this validation alone does not determine whether the difference in gene numbers reflects actual changes in the HB repertoire of the two subspecies. Differences in genome assemblies, annotation quality and methodology, and gene model prediction may also contribute to the observed discrepancies. Therefore, the difference between *indica* and *japonica* should be interpreted with caution and not necessarily as direct evidence of *HB* gene expansion or loss.

In our analysis of putative *cis*-elements located in the promoter regions of *HB* genes (**Figure 4**), ABRE was identified as the most abundant regulatory motif. This element has been directly associated with abscisic acid (ABA) signaling, a key phytohormone involved in regulating multiple physiological processes, including stomatal closure, induction of seed and bud dormancy, and activation of tolerance mechanisms against abiotic stresses such as drought, cold, and salinity (Wei et al., 2025). In addition to regulating water balance, ABA promotes the accumulation of protective cellular compounds, including chaperones, LEA proteins, antifreeze proteins, sugars, and proline. It also activates detoxification pathways that adjust redox balance and modulate ion transport, thereby facilitating the recovery of cellular homeostasis and enhancing stress tolerance. Under drought conditions, ABA levels can increase up to 50-fold, which is why it is widely regarded as a stress hormone (Gómez-Porras et al., 2007; Li et al., 2017). In *Arabidopsis*, rice, and soybean, ABRE has been shown to be the most conserved sequence in dehydration-inducible promoters, suggesting that ABRE-dependent transcriptional regulation is a conserved mechanism across these species. In contrast, cold-inducible promoters exhibit more diverse sequences, indicating that cold-responsive regulation may vary among species (Maruyama et al., 2012). We also retrieved a high number of occurrences of the CGTCA-motif and TGACG-motif, which correspond to *cis*-regulatory elements associated with the methyl jasmonate (MeJA) response. This compound is the methyl ester of jasmonic acid (JA), a volatile component isolated from jasmine (*Jasminum grandiflorum* L.) oil (Wang et al., 2020b; Joulain, 2021; Saadati and Saffari, 2026). MeJA acts as a signaling phytohormone that modulates gene and protein networks enabling plants to respond to diverse biotic and abiotic stresses; it also activates defense mechanisms and regulates growth and development, marking it as a central component of plant stress signaling pathways (Ali and Baek, 2020). Studies in pepper (*Capsicum annuum* L.) have shown that CaCIPK3 acts as a positive regulator of drought tolerance by promoting MeJA signaling and strengthening the antioxidant system, whereas its silencing increases sensitivity to osmotic stress and MeJA (Ma et al., 2021). Similarly, in rice, jasmonic acid and its derivatives have been shown to be essential for basal immunity against pathogens by promoting the expression of defense genes and enhancing resistance during infection (Ma et al., 2022). Furthermore, our bioinformatic analyses predicted 98 ARE (anaerobic responsive element) motifs, which are frequent and diverse in *Arabidopsis*, including sequences such as TTCCCTGTT, (A/C/G)AAAAACAAA, and AGCAGC, present in promoter regions of genes related to metabolism, signaling, growth, and stress responses. Plant evolution appears to have reduced both the number and effectiveness of AREs, likely as an adaptation to increased atmospheric oxygen levels (Sadeghnezhad et al., 2014).

To better understand differences in transcriptional regulation of each *HB* gene, *de novo* motif discovery was performed on the corresponding promoter sequences (**Figures 5**–**9**). Overrepresented motifs were predicted in the promoters of all *HB* subfamilies. The *HB* promoters contained motifs potentially linked with responses to desiccation, salinity and cold (Verma and Bhatia, 2019); osmotic stress (Li et al., 2021); drought, extreme temperatures and salinity (Liu et al., 2022); cold, heat, salinity, and drought (Patel et al., 2024); as well as ethylene and extreme environments (Duan et al., 2026). Thus, our *de novo* survey confirmed the likely conservation of regulatory networks within this family of transcription factors. However, the *cis*-regulatory elements predicted need experimental testing using high-throughput, high-resolution, and functional validation technologies. The most cutting-edge technologies to achieve this aim include massively parallel functional assays, direct genome editing, and single-cell single-molecule resolution approaches (Lau and Suh, 2018; La Fleur et al., 2024; DeGroat and Kreimer, 2026; Agarwal et al., 2025).

To further explore the biological importance of *HB* genes, we utilized the eFP-Plant platform for the visual analysis, interpretation, and analysis of large-scale, publicly available plant transcriptome datasets (Winter et al., 2007; Sullivan et al., 2025). While these retrieved expression profiles were not independently verified via quantitative RT-PCR (qRT-PCR) in the current study, modern sequencing and microarray platforms feature high, well-documented accuracy, and our data passed strict quality control and false discovery rate corrections. Furthermore, numerous published studies demonstrate a strong correlation between this profiling method and qRT-PCR (*e.g.*, Ortiz-Ramírez et al., 2016; Sullivan et al., 2025; Chen et al., 2026), supporting our focus on a broad computational model and large-scale trends rather than individual gene expression changes. While the lack of qRT-PCR analysis represents a study limitation this rigorous *in silico* analysis serves as a foundational framework for our ongoing and future wet-lab experiments, which are currently being planned to validate individual gene expressions using qRT-PCR, RNA-Seq, and GWAS.

The *in-silico* gene expression findings suggest potential responsiveness of *HB* genes to various abiotic stress factors (**Figure 10**) and tissue specificity (**Figure 11**). The genes *LOC_Os11g03420* (ZF-HD) and *LOC_Os05g48990* (WOX) were predicted to be induced under water deficit. Studies in sunflower (*Helianthus annuus* L.) have shown that members of the ZF-HD subfamily contribute to drought tolerance; in particular, *HaZF3* exhibited early induction under drought conditions based on RNA-seq and qRT-PCR analyses, demonstrating its role in adaptive responses to water deficit (Zhang et al., 2025b). Similarly, studies in rye (*Secale cereale* L.) have reported that several ZF-HD subfamily members, including ScZHD1, ScZHD3, ScZHD5, ScZHD6, ScZHD7, ScZHD9, ScZHD12, ScMIF5, ScMIF6, and ScMIF7, are induced under drought stress, highlighting the key regulatory role of these factors in adaptation to water deficit (Li et al., 2025a). The WOX subfamily member WOX11 contributes to drought tolerance in rice by regulating OsPRX130, thereby promoting root growth and adaptation to water deficit (Deng et al., 2025; Tan et al., 2025). Consistently, our *in-silico* analyses also showed induction of *LOC_Os06g12400* (HB-PHD), *LOC_Os03g03260* (TALE), and *LOC_Os03g12860* (HD-ZIP) under salinity conditions. Studies in wheat have demonstrated that TALE subfamily genes such as *TaKNOX11-A* are induced under salt stress and confer tolerance when overexpressed in *Arabidopsis*, reducing oxidative damage and increasing proline accumulation (Han et al., 2022). Likewise, induction of the maize *HD-Zip I* gene *Zmhdz10* confers enhanced drought and salinity tolerance by activating ABA-dependent signaling pathways, reducing oxidative damage, and promoting proline accumulation (Zhao et al., 2014). We also observed moderate induction of *LOC_Os11g03420* (ZF-HD) expression under cold conditions. Similarly, wheat *TaZF-HD* genes have been reported to be induced under salinity, drought, and cold stress (Liu et al., 2021a).

In general, transcription factors such as HB function through complex crosstalk involving regulatory cascades and protein-protein interactions, which can modulate the expression of downstream stress-responsive genes (Bharti et al., 2026). In rice, a MYB transcription factor OsMYBR57 physically interacts with a homeodomain TF OsHB22. This OsMYBR57-OsHB22 module synergistically regulates expression of bZIP transcription factor-encoding genes *OsbZIP66* and *OsbZIP72*, which in turn tune expression of drought-responsive genes *OsLEA3* and *Rab21* (Yang et al., 2022).

Our gene expression results across different rice developmental stages revealed potential functional diversification of *Homeobox* genes, suggesting key roles in regulating phenological processes. In particular, *LOC_Os03g03260* (TALE) showed strong inducible expression in mature leaves, suggesting its possible involvement in leaf differentiation and development in rice. This is consistent with studies showing that TALE proteins (KNOX and BEL1-like) regulate shoot apical meristem function and the morphogenesis of leaves, stems, and inflorescences (Hamant and Pautot, 2010; Di Giacomo et al., 2013). In a similar fashion, TALE analyses in sorghum have shown preferential expression in leaves and stems, indicating their importance in vegetative development (Yao et al., 2024). Higher relative expression of *LOC_Os11g03420* (ZF-HD), *LOC_Os03g12860* (HD-ZIP), *LOC_Os05g48990* (WOX), and *LOC_Os06g12400* (HB-PHD) was also observed during seed development, suggesting their potential involvement in physiological processes related to maturation and differentiation of reproductive tissues. Previous studies have highlighted that transcription factors such as WOX and HD-ZIP regulate critical events in embryogenesis and maturation through combinatorial networks controlling seed-specific gene expression, underscoring their relevance and potential roles in seed development (Verma et al., 2022).

We are currently performing further biochemical, physiological and molecular analysis to demonstrate the role of the HB transcription factors in different rice genotypes in response to osmotic stress.

## 5 Conclusions

Our results suggest that the Homeobox (HB) transcription factor family plays a vital role in regulating tissue development and osmotic stress tolerance in rice, particularly against drought, salinity, and cold. By analyzing 268 initial sequences from *Oryza sativa* subspecies *indica* and *japonica*, we filtered out redundant isoforms to identify 223 non-redundant HB genes distributed across all 12 chromosomes. Phylogenetic analysis classified these genes into six major subfamilies: HB-PHD, HD-ZIP, TALE, WOX, ZF-HD, and HB-OTHERS. *In silico* expression profiles predicted tissue- and developmental-stage-specific activity, while promoter analysis revealed 688 putative *cis*-regulatory elements potentially tied to hormonal and stress signaling pathways, such as ABA and MeJA (notably ABRE, CGTCA/TGACG-motifs, and ARE). Ultimately, this comprehensive structural and functional profile establishes a foundation for targeted methodologies to potentially enhance crop resilience, likely supporting food security and sustainable agriculture under a changing climate. Cutting-edge approaches including synthetic biology and CRISPR gene editing for agricultural efficiency may help support climate-smart and sustainable agriculture, in line with Sustainable Developments Goals.

## Data Availability

The datasets presented in this study can be found in online repositories. The names of the repository/repositories and accession number(s) can be found in the article/**Supplementary Material**.
